# Isotope systematics and chemical composition of tin ingots from Mochlos (Crete) and other Late Bronze Age sites in the eastern Mediterranean Sea: An ultimate key to tin provenance?

**DOI:** 10.1371/journal.pone.0218326

**Published:** 2019-06-26

**Authors:** Daniel Berger, Jeffrey S. Soles, Alessandra R. Giumlia-Mair, Gerhard Brügmann, Ehud Galili, Nicole Lockhoff, Ernst Pernicka

**Affiliations:** 1 Curt-Engelhorn-Zentrum Archäometrie gGmbH, Mannheim, Germany; 2 Department of Classical Studies, University of North Carolina, Greensboro, NC, United States of America; 3 AGM Archeoanalisi, Merano, BZ, Italy; 4 The Zinman Institute of Archaeology, University of Haifa, Haifa, Israel; 5 Institut für Geowissenschaften, Ruprecht-Karls-Universität Heidelberg, Heidelberg, Germany; Universita degli Studi di Milano, ITALY

## Abstract

The origin of the tin used for the production of bronze in the Eurasian Bronze Age is still one of the mysteries in prehistoric archaeology. In the past, numerous studies were carried out on archaeological bronze and tin objects with the aim of determining the sources of tin, but all failed to find suitable fingerprints. In this paper we investigate a set of 27 tin ingots from well-known sites in the eastern Mediterranean Sea (Mochlos, Uluburun, Hishuley Carmel, Kfar Samir south, Haifa) that had been the subject of previous archaeological and archaeometallurgical research. By using a combined approach of tin and lead isotopes together with trace elements it is possible to narrow down the potential sources of tin for the first time. The strongly radiogenic composition of lead in the tin ingots from Israel allows the calculation of a geological model age of the parental tin ores of 291 ± 17 Ma. This theoretical formation age excludes Anatolian, central Asian and Egyptian tin deposits as tin sources since they formed either much earlier or later. On the other hand, European tin deposits of the Variscan orogeny agree well with this time span so that an origin from European deposits is suggested. With the help of the tin isotope composition and the trace elements of the objects it is further possible to exclude many tin resources from the European continent and, considering the current state of knowledge and the available data, to conclude that Cornish tin mines are the most likely suppliers for the 13^th^–12^th^ centuries tin ingots from Israel. Even though a different provenance seems to be suggested for the tin from Mochlos and Uluburun by the actual data, these findings are of great importance for the archaeological interpretation of the trade routes and the circulation of tin during the Late Bronze Age. They demonstrate that the trade networks between the eastern Mediterranean and some place in the east that are assumed for the first half of the 2^nd^ millennium BCE (as indicated by textual evidence from Kültepe/Kaneš and Mari) did not exist in the same way towards the last quarter of the millennium.

## 1. Introduction

**Fig 1:**
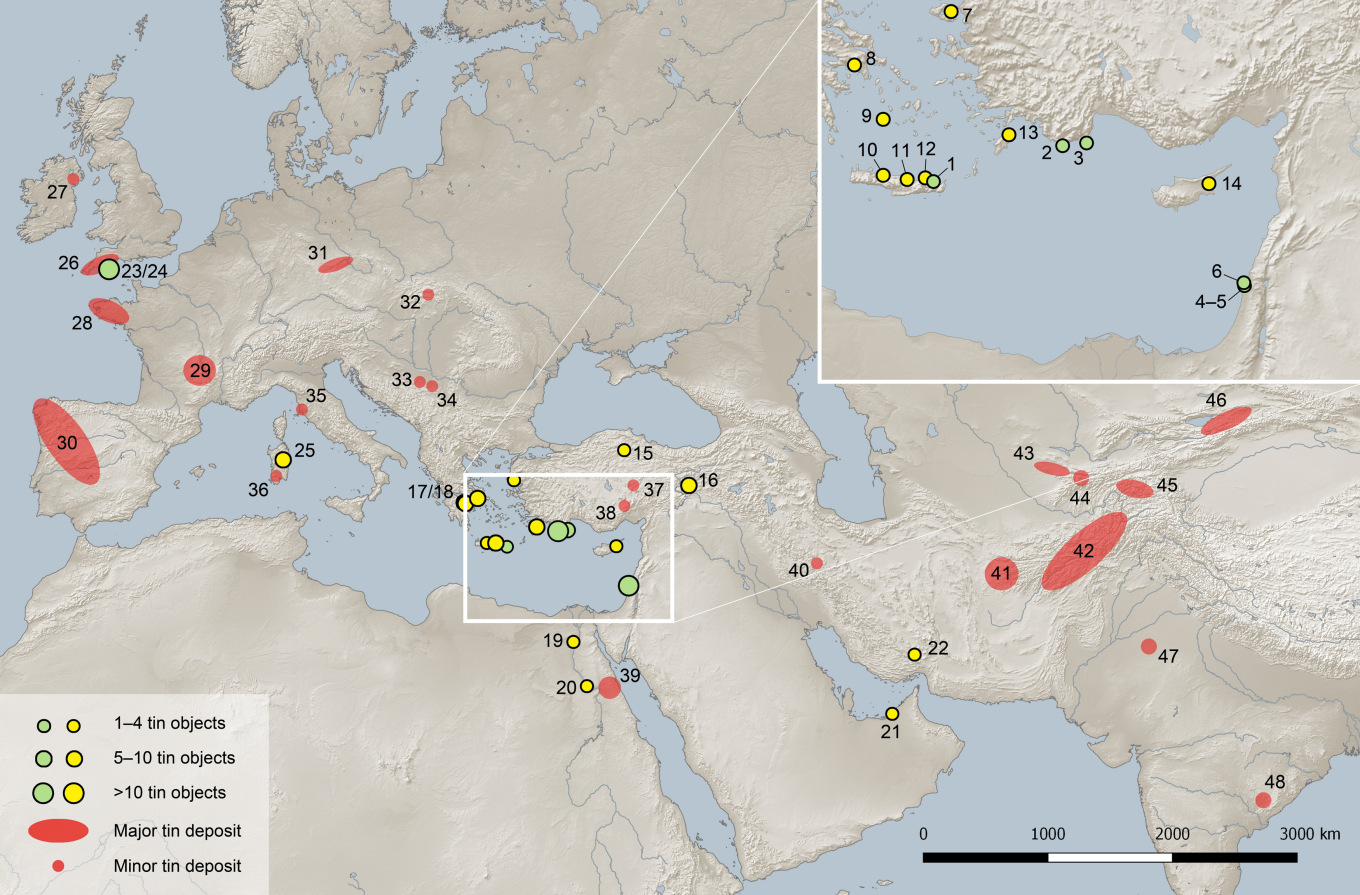
Map of Eurasia showing the locations of the tin ingots mentioned in the text (green dots), other tin objects in the eastern Mediterranean and the Near East before 1000 BCE (yellow dots) and major and minor tin deposits. 1: Mochlos (Crete), Greece, 2: Uluburun, Turkey, 3: Gelidonya, Turkey, 4: Hishuley Carmel, Israel, 5: Kfar Samir south, Israel, 6: Haifa, Israel, 7: Thermi (Lesbos), Greece, 8: Athens, Greece, 9: Phylakopi (Milos), Greece, 10: Rethymno (Crete), Greece, 11: Knossos (Crete), Greece, 12: Kalydon (Crete), Greece, 13: Ialysos (Rhodos), Greece, 14: Salamis (Cyprus), Turkey, 15: Alaca Höyük, Turkey, 16: Tülintepe, Turkey, 17: Mycenae, Greece, 18: Dendra, Greece, 19: Abydos, Egypt, 20: Gurob, Egypt, 21: Tell Abraq, United Arab Emirates, 22: Tepe Yahya, Iran, 23: Salcombe, United Kingdom, 24: Erme Estuary, United Kingdom, 25: S’Arcu e is Forros, Sardinia, Italy, 26: Cornwall/Devon, United Kingdom, 27: Mourne Mountains, Down County, North Ireland (United Kingdom), 28: Brittany, France, 29: Massif Central, France, 30: North Portugal/Spain, 31: Erzgebirge province with the Bohemian-Saxon Erzgebirge, Vogtland, Fichtelgebirge, Kaiserwald (Slavkovský les), 32: Slovak Ore Mountains, Slovak Republic, 33: Mt. Cer, Serbia, 34: Mt. Bukulja, Serbia, 35: Monte Valerio, Italy, 36: Sardinia, Italy, 37: Kestel, Turkey, 38: Hisarcık, Turkey, 39: Eastern Desert, Egypt, 40: Deh Hosein, Iran, 41: Western Afghanistan (Herat and Farah provinces), 42: Central/north-eastern Afghanistan (Hindu Kush), 43: Karnab/Lapas/Čangali (Zeravšan valley), Uzbekistan, 44: Mušiston/Takfon (Hissar Mountains), Tadzhikistan, 45: Pamir, Tadzhikistan, 46: Kyrgyzstan, 47: Tosham, Bhiwani district, India, 48: Bastar district/Koraput district, India, 49 (not on the map): Kazakhstan. Size of green and yellow symbols on the inset map do not correlate with number of objects as on the main map (map: D. Berger, C. Frank using Natural Earth geo data and QGIS Geographic Information System. QGIS Development Team, 2019. Open Source Geospatial Foundation. http://qgis.org).

Tin objects are extremely rare in the archaeological record, and only very few are known from prehistoric contexts (for artefacts in the eastern Mediterranean and the Near East dating from before 1000 BCE see Fig 1; summary of Eurasian finds in [1]). This is probably due to a number of reasons. Unalloyed tin corrodes easily in a damp environment in which corrosion stimulators such as chlorides or sulphates are present (for example at the seaside) [2–4]. Deterioration may be enhanced at low temperatures, less than 13°C, when the crystal structure of tin changes, turning the white metal to a grey powder. This so-called tin pest is often stated in archaeological literature [5–8], but since its occurrence has not yet been confirmed on prehistoric artefacts its contribution to the problem is certainly small. Because of this and because corrosion does not make objects simply disappear, socio-economic factors and the predominant usage of tin for the production of bronze are the more likely explanations for the general rarity of ancient tin objects.

Tin ingots, the subject of this paper, are a special group of artefacts. They represent a specific type of trade goods, and a small number of them, dating from the Late Bronze Age (LBA), were discovered in the eastern Mediterranean area (Table 1 and Fig 1). One rare example, and to date the only one from a terrestrial context in the whole Mediterranean region, is the tin ingot from Mochlos (Fig 1). The Minoan settlement is located on a small island very close to the north-eastern coast of Crete. The island was connected to the Cretan mainland through a land bridge that was exposed until Hellenistic times. The site was an important commercial centre throughout the Bronze Age (BA), but in particular during the Neopalatial period (1700–1425 BCE). It had rich metal and pottery traditions, was an important trading port along the routes to and from Cyprus and the Levant, and was also a religious centre [7; 9]. It was destroyed by earthquakes in the Neopalatial period, especially at the time of the Santorini eruption (around 1530 BCE) when a large number of buildings had to be rebuilt and the metal and pottery workshops were moved to the coast of the Cretan mainland [10–11].

**Table 1:** Compilation of LBA tin ingots from the eastern Mediterranean and related information.

Site	Quantity	Dating	Dating quality	Context	Circumstances of finding	Museum	References
Mochlos (GR)	1	1530–1425 BCE	Secure	Storeroom 1.7 inside building B.2	Archaeological excavation	AMA	[6–7; 12]
Uluburun (TR)	160	*ante* 1318 BCE	Secure	Shipwreck	Archaeological excavation	MUA	[5; 14–18; 32–33; 35, 62, 99; 155]
Gelidonya (TR)	8 kg	~1200 BCE	Secure	Shipwreck	Archaeological excavation, nature of tin unclear (‘whitish material’)	MUA	[19–20; 156]
Hishuley Carmel (IL)	15	ca. 1300 BCE	Unsecure	Shipwreck	Archaeological excavation	NMM	[16; 23–27; 31–33; 35; 72–73]
Kfar Samir south (IL)	10	14^th^–13^th^ c. BCE	Secure	Shipwreck	Archaeological survey	NMM	[24–25; 27; 31–34; 72–73]
Haifa (IL)	30	ca. 1300 BCE	Unsecure	Shipwreck?	Discovered by the fisherman Adib Shehade	NMM, EIM	[21; 28–31; 35; 52]

AMA, Archaeological Museum, Agios Nikolaos, Greece; EIM, Eretz Israel Museum, Tel Aviv, Israel; NMM, The National Maritime Museum, Haifa, Israel; MUA, Bodrum Museum of Underwater Archaeology, Turkey.

**Fig 2:**
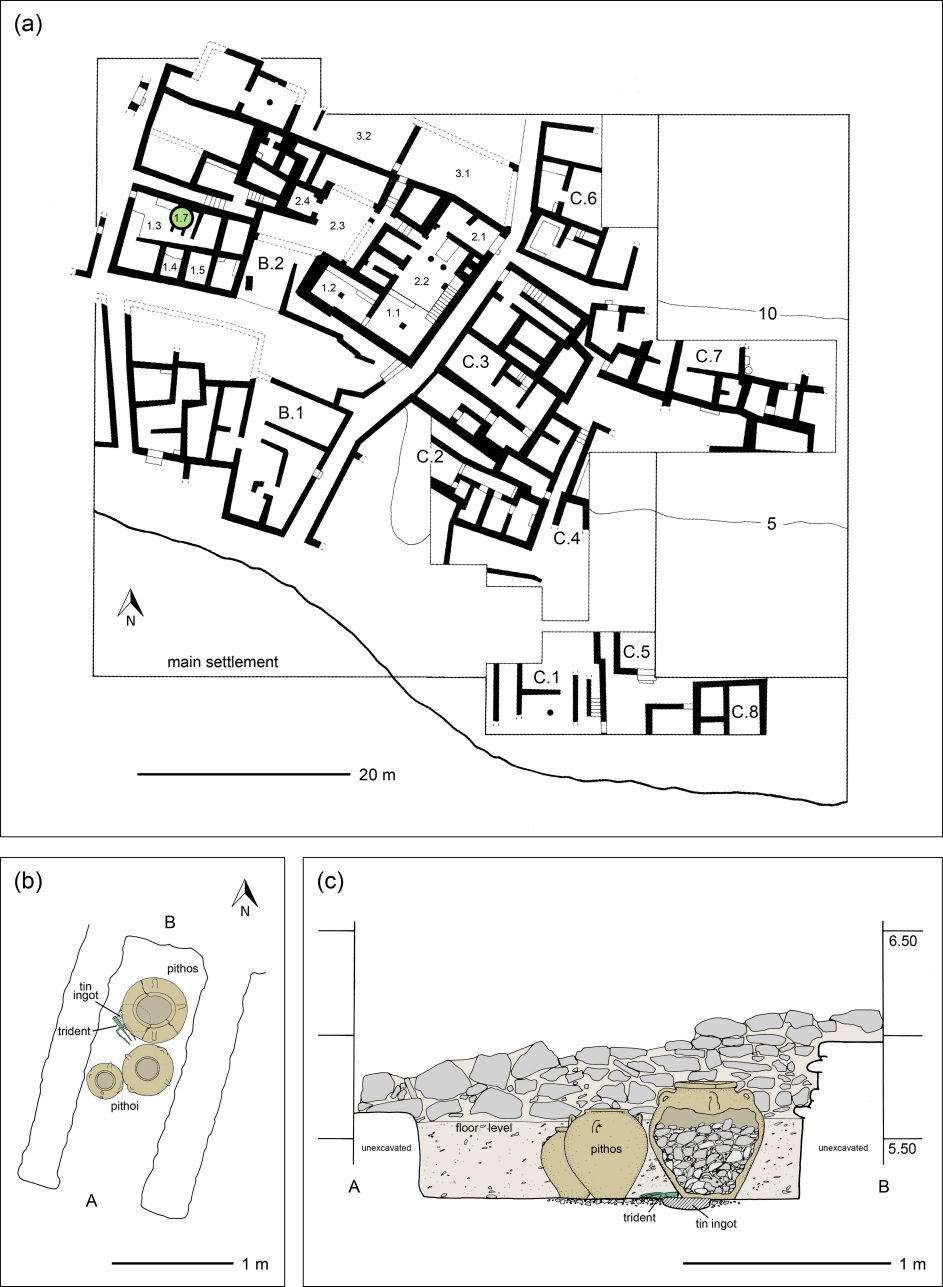
**Map of part of the main settlement of Mochlos with the find location of the tin ingot in storeroom 1.7 (a).** Details of the archaeological context inside the storeroom is shown in (b) and a section in north-south direction in (c) (images: modified and reprinted from [12] under a CC BY license, with permission from the INSTAP Academic Press, original copyright 2007).

**Fig 3:**
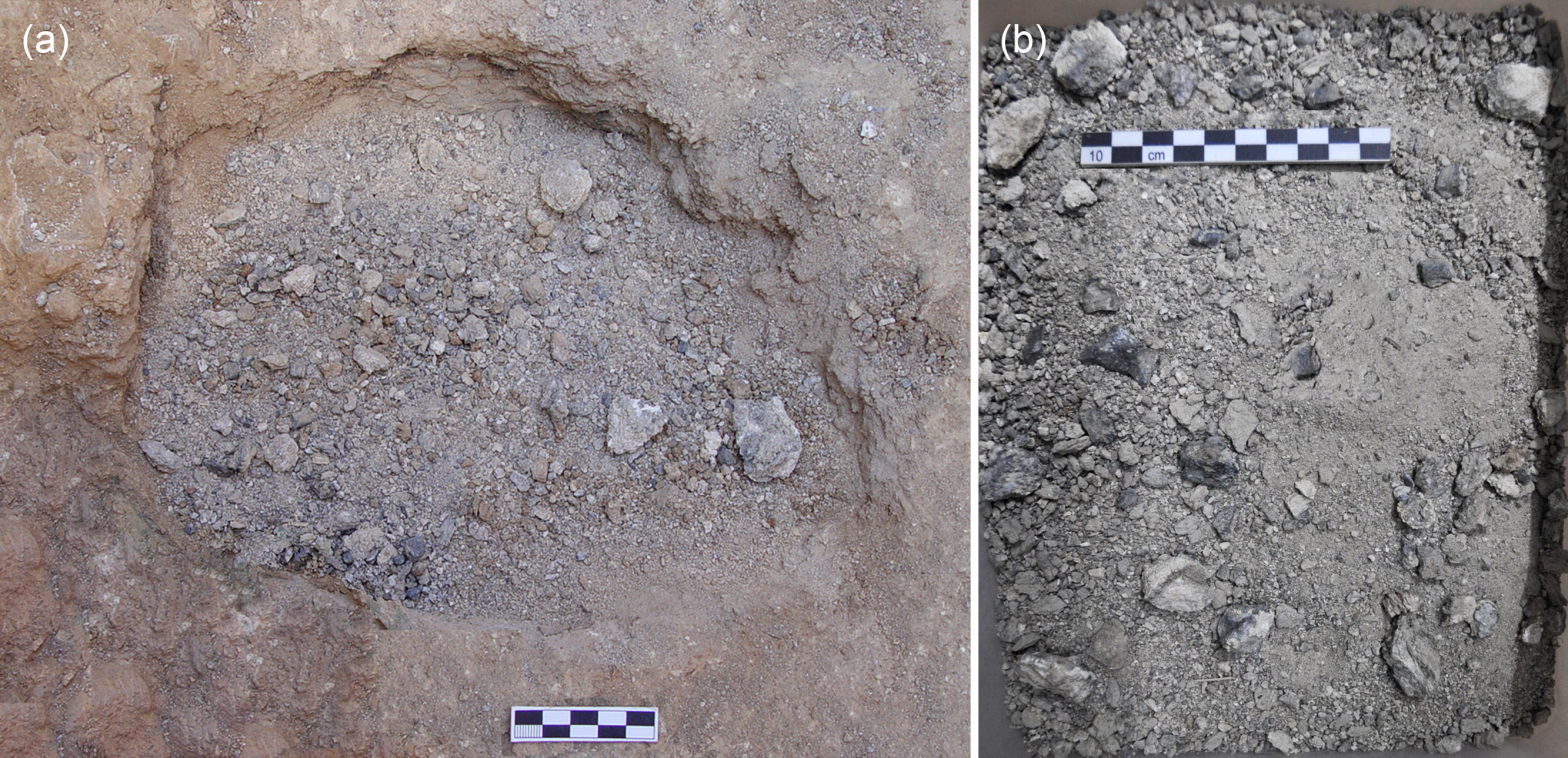
**The tin ingot from Mochlos on site (a) and close-up view (b) illustrating its disintegrated condition.** The original shape of the ingot could only be reconstructed by the discoloration of the soil (photos: J.S. Soles).

In 2004, during an excavation in the Mochlos settlement the tin ingot was unearthed in a storeroom belonging to the western wing of a large ceremonial building [7, 12–13]. This building–designated B.2 –had many rooms, and next to the storeroom (1.7) with the tin ingot was a large room (1.3), presumably used for a drinking ceremony (Fig 2A). On the opposite side, there was another space (1.4) in which six bronze basins were found [13]. Inside the storeroom 1.7 itself three pithoi were buried in the ground, so that their mouths were just above floor level, a common practice in Minoan houses to store food or beverages. Beneath the largest and innermost pithos, ca. 0.4 metres below ground level, the now completely disintegrated tin ingot was located next to a bronze trident (Fig 3). It had been placed together with the trident before the pithoi were positioned and the earth filled up to the original floor level (Fig 2B and 2C). The tin ingot belonged to a precious foundation deposit that was offered to the goddess to whom the building was dedicated and was protected by the trident. As part of a foundation deposit it was laid in place when the building was constructed at the beginning of the Late Minoan IB period, ca. 1530 BCE (*terminus ante quem*), and lay hidden when the building was destroyed a hundred years later. It is approximately 200 years older than the other ingots discussed in this paper (Table 1).

Tin ingots have been recovered more frequently from underwater contexts (Table 1). The best-known examples are the LBA finds from the wreck of the Uluburun ship discovered off the coast of Turkey in 1982 [14–17; 18], which sank shortly before 1318 BCE (Fig 1). In addition to 10 tons of copper ingots, the cargo contained glass ingots, faience and resin, objects made of gold, silver, ivory and amber and, strikingly, one ton of tin. Among the finds, there was also a bronze trident representing the closest typological parallel to the trident found at Mochlos [14]. The unique tin cargo itself comprises ca. 160 ingots of different shapes, including such of oxhide shape, and four finished tin artefacts. The tin ingots was most likely intended to be alloyed with the copper on board, but which port it was destined for and where the tin came from is still an unsolved problem. Pulak [18] argues for an east-west Mediterranean searoute with the homeport having been situated along the northern Israeli Carmel or southern Lebanon coast.

A second shipwreck from around 1200 BCE with a large cargo had been discovered a few years earlier off Cape Gelidonya, Turkey (Fig 1). In addition to raw products, finished objects and a folded tin foil, Bass [19] documented several kilograms of a whitish material that was considered a corrosion product of tin by Dykstra [20]. However, Maddin et al. [21] and Charles [22] challenged this interpretation because the material contained mainly calcium (71% as CaCO_3_) and only a small amount of tin (ca. 14% as SnO). Therefore, some scholars hypothesised that the material might be cassiterite ore that was designed to be mixed with metallic copper [22]. Since then, no other analyses seem to have been carried out, so it is still not clear whether the Gelidonya ship actually carried tin or not. It is also unknown which route the ship took and where the goods came from.

**Fig 4:**
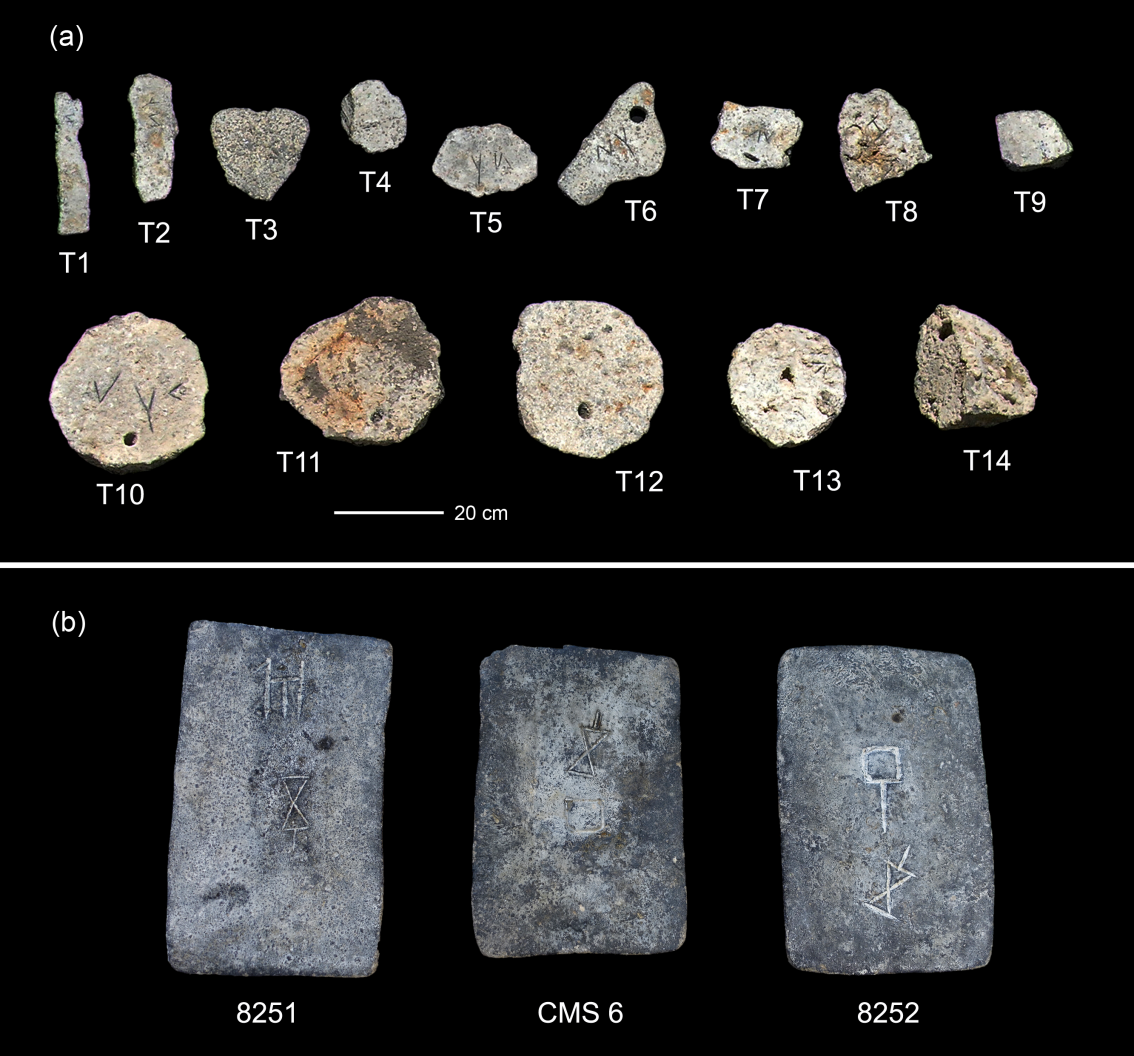
Metal cargos of the alleged ships that wrecked offshore the Israeli coast. (a) Tin ingots from Hishuley Carmel, part of them with Cypro-Minoan marks; numbering corresponds to the original sample designation in Table 3. (b) Three out of 30 tin ingots from Haifa with Cypro-Minoan inscriptions with their original label from the literature. Scale applies to all ingots on the figure (photos: E. Galili, Fig 4A modified and reprinted from [26] under a CC BY license, with permission from the International Journal of Nautical Archaeology, original copyright 2013).

The latter also applies to a group of 15 tin ingots recovered in four campaigns from an alleged shipwreck at the coast of Hishuley Carmel, Israel (Figs 1 and 4A), together with two oxhide copper ingots and several stone anchors [23–27]. Because the archaeological context was missing, the exact dating of the finds is uncertain, but ‘Cypro-Minoan’ symbols inscribed on the surface of several ingots suggest a LBA date of around 1300 BCE [23–24; 26]. For the same reason, Maddin et al. [21] and Stech-Wheeler et al. [28] assigned two rectangular tin ingots found off the Israeli coast near Haifa to the LBA (Fig 4B, 8251 and 8252). Their hypothesis was questioned by Artzy [29], however, who reported on two very similar ingots from Israel (in the literature the place where they were found is mistakenly called Dor or Atlit) with ‘Cypro-Minoan’ inscriptions (Fig 4B, CMS 6). The upper surface of one of the ingots carries the conjectured head of *Arethusa* (a Greek fountain goddess); therefore, in her opinion, all four objects should be dated to the 5^th^ century BCE. However, careful inspection on the *Arethusa* head by one of the authors (EG) suggested that this image is a random metal spill and was not produced on purpose. In addition, recent investigations (unpublished information) proved the four ingots to belong to the same assemblage. They are the remains of a set of originally 30 rectangular tin ingots (with trapezoidal cross section) that was found in the 1970s by a fisherman (Adib Shehade) offshore Kfar Samir, Israel (Table 1) [30]. The ingots were later sold by the fisherman to a tinsmith who used the tin to repair car radiators. From the set, the surviving four ingots were bought from the tinsmith on behalf of the University of Haifa. Further inquiries revealed that the ingots were retrieved some 60 metres north of another underwater site (the Kfar Samir north), which yielded several broken copper (oxhide) and lead ingots [25]. However, although found relatively close to that site, the rectangular tin ingots may have belonged to a separate shipwreck. The exact context of the tin ingots is still uncertain though because the site was not surveyed with archaeological methods. In the literature, several find locations were specified for these ingots (Haifa, Dor, Atlit), and even though we are aware of the exact location now, we use ‘Haifa’ here so as not to produce further confusion by introducing a new location. Dor [31] and Atlit [32–33] lie farther south of Haifa and are definitely not the correct locations.

In addition to these 45 raw products, another ten tin ingots exist from an off-shore site near Kfar Samir in Israel (called Kfar Samir south). They are also thought to belong to the LBA, and to date from ca. 14^th^–13^th^ century BCE [25; 27; 34]. They were salvaged together with Egyptian stone anchors, bronze objects, a bronze sickle sword and five lead ingots during an underwater survey of a shipwreck just 900 metres north of the Hishuley Carmel and 550 metres south of the ‘Haifa’ site (Fig 1). As with the anchors, some of the ingots have inscriptions. The cargo assemblage of this wreck is assumed to be of Egyptian provenance [34], whereas the Hishuley Carmel objects may be associated with Cyprus or the Syro-Palestinian coast [24, 35]. In summary, presently some 215 tin ingots weighing almost one and a half tons are known from BA or presumed BA contexts. In this paper we investigate this material group with the most modern scientific facilities in order to elucidate their history and the provenance of the tin.

## 2. Previous investigations of tin ingots

Because of their rarity, it is hardly surprising that many of the ingots listed above have been analysed in the past, some even a couple of times, by various research groups (Table 2). The primary aim was always to unveil the origin of the tin, but questions regarding trade routes also arose since tin had to be transported and traded over long distances because of insignificant cassiterite resources in the Mediterranean World. Despite a number of tin-containing minerals (such as stannite, mushistonite and kësterite), cassiterite was the only economically usable tin ore mineral in prehistoric times. With the exception of the disputed tin occurrences at Kestel and Hisarcık, Turkey [36–41] and small mineralisations on Sardinia and at Monte Valerio, Italy [42–46], there are no large-scale exploitable tin deposits in the vicinity of the places where the tin ingots were found (Fig 1). The problem of the cassiterite sources also applies to the great many BA bronzes from the eastern Mediterranean region and the Near East [47–51].

**Table 2:** Archaeometallurgical studies and kind of analyses that had been carried out on LBA tin ingots in the past.

Site	Study	Chemical analysis		Lead isotope analysis		Tin isotope analysis		Metallography
		Done	Analytical method	Done	Analytical method	Done	Analytical method	
Mochlos	This study	+	LA-Q-ICP-MS, SEM-EDX	+	MC-ICP-MS	+	MC-ICP-MS	+
Uluburun	[5]	+	AAS	–		–		+ (mention)
	[16]	–		+	TIMS	–		–
	[62]	+	NAA, MSID	+	TIMS	–		–
	[155]	–		+	TIMS	–		–
	[99]	+	ICP-MS	–		–		+ (mention)
	[32]	–		–		+	MC-ICP-MS	–
	[73]	–		–		+	MC-ICP-MS	–
	[33]	–		–		+	MC-ICP-MS	–
	[26]	(+)	(NAA, MSID)	+	TIMS	–		–
	This study	+	LA-Q-ICP-MS	+	MC-ICP-MS	+	MC-ICP-MS	+
Gelidonya	[20]	+	AAS?	–		–		–
Hishuley Carmel	[24]	+	NAA	–		–		–
	[31]	+	INAA	–		+	TIMS	–
	[16]	–		+	TIMS	–		–
	[62]	+	NAA, MSID	+	TIMS	–		–
	[72]	–		–		+	MC-ICP-MS	–
	[32]	–		–		+	MC-ICP-MS	–
	[73]	–		–		+	MC-ICP-MS	–
	[33]	–		–		+	MC-ICP-MS	–
	[26]	(+)	(NAA, MSID)	+	TIMS	–		–
	This study	+	Q-ICP-MS	+	MC-ICP-MS	+	MC-ICP-MS	–
Kfar Samir south	[31]	+	INAA	–		+	TIMS	–
	[62]	+	NAA, MSID	+	TIMS	–		–
	[72]	–		–		+	MC-ICP-MS	–
	[32]	–		–		+	MC-ICP-MS	–
	[73]	–		–		+	MC-ICP-MS	–
	[33]	–		–		+	MC-ICP-MS	–
	This study	+	Q-ICP-MS	+	MC-ICP-MS	+	MC-ICP-MS	–
Haifa	[21]	+	AAS, AES, INAA	–		–		+
	[52]	+	AAS	–		–		–
	[31]	+	INAA	–		+	TIMS	–
	This study	+	Q-ICP-MS	+	MC-ICP-MS	+	MC-ICP-MS	–

AAS, atomic absorption spectrometry; AES, atomic emission spectrometry; MSID, mass spectrometric isotope dilution; (I)NAA, (instrumental) neutron activation analysis; (LA-)Q-ICP-MS, (laser ablation) quadrupole mass spectrometry; SEM, scanning electron microscopy; EDX, energy dispersive X-ray spectrometry; TIMS, thermal ionisation mass spectrometry; +, analysis was carried out;–, analysis was not performed.

Geographically not too distant tin deposits of significant scale are located in the eastern Desert of Egypt (Fig 1), but they do not seem to have been exploited in prehistoric times [48, 51–52]. Large mineralisations in western and central Europe, such as those in Cornwall/Devon, United Kingdom, in Brittany and the Massif Central, France, the Iberian peninsula and the Saxon-Bohemian tin province with the Erzgebirge (*Krušné hory*), the Fichtelgebirge, the Vogtland and the Kaiserwald (Slavkovský les) have been suggested as possible sources for cassiterite used for Mediterranean and Near Eastern tin-bearing objects. However, the large deposits in central Asia, especially in Afghanistan, are currently considered the most likely sources of tin. This view is mainly based on text documents, rare trade goods such as lapis lazuli and lead isotope data of bronzes [47–48; 53–55]. Apart from some weak indications [56], compelling archaeological evidence for the exploitation of tin ores in Afghanistan, however, is still lacking [57]. On the other hand, ^14^C dates from prehistoric workings indicate active tin mining in Uzbekistan, Tadzhikistan or Kazakhstan during the late 3^rd^ and the 2^nd^ millennia BCE [58–61]. However, direct relationships of tin and bronze artefacts from the Eastern Mediterranean region and the Near East could not yet be established with these tin ores.

So far, chemical analyses of the ingots from Uluburun, Hishuley Carmel, Kfar Samir south and Haifa have not provided suitable fingerprints unveiling the provenance of the tin (for references *cf*. Table 2). This is mainly because unalloyed tin is commonly quite pure with only a few trace elements partitioning to the metal phase during the smelting of tin ores [62–64]. Several studies determined lead isotope ratios of the tin ingots from the Mediterranean area (for references *cf*. Table 2), but since the tin ore–and also the tin–usually contains very low lead concentrations of less than ca. 100 μg g^-1^ contaminations with lead from the smelting structures, fuel or aggregates could easily modify the isotope signature [65]. Accordingly, conclusions about the provenance which are based only on the lead isotope ratios are ambiguous if lead contamination cannot be excluded.

It is therefore more advantageous to use the isotope composition of the main constituent of the ingots, i.e. the tin itself. Recent studies have confirmed that the tin isotope composition of ores and metals is of great value for the sourcing of tin and the establishment of relationships between artefacts [1; 66–71]. The pioneering studies on tin isotopes carried out on some tin ingots from Hishuley Carmel, Kfar Samir south, Haifa and Uluburun have already revealed similarities and differences in the isotope composition [31–33; 72–73], but no conclusions could be drawn on the origin of the tin in those studies because of the lack of ore data. Only a dozen ores from very different locations were characterised in those days.

In this paper we intend to follow up the approach of the early studies by presenting new tin and lead isotope data of tin ingots and by comparing them with an enlarged data base of tin ores. Almost all ingots from the eastern Mediterranean that have been previously studied are reconsidered here (Table 2), and the older data is critically reviewed. At the same time, we investigate the Mochlos tin ingot in more detail since this has not been done before. This involves metallographic examination and analyses with scanning electron microscopy and energy-dispersive X-ray spectroscopy (SEM-EDX) as well as X-ray diffractometry (XRD). The study is completed by the determination of the chemical composition of many tin ingots. The ultimate goal of this combined approach is once again to unravel the history and provenance of the tin in the ingots.

## 3. Materials and methods

Tin ingots from the above-mentioned sites, with the exception of the Gelidonya shipwreck, were chosen for the present study and analysed in the laboratory of the Curt-Engelhorn-Zentrum Archäometrie Mannheim, Germany (CEZA). This includes 14 out of 15 ingots of the Hishuley Carmel wreck, seven of a total of ten of the Kfar Samir south wreck and two of the supposed shipwreck off the coast of Haifa, all of which consisted of well-preserved tin metal. Three ingots of the Uluburun wreck (with two samples from the same ingot KW 203) were also examined, but they were entirely corroded. As compiled in Table 3, almost all samples were analysed previously regarding their chemical and lead and tin isotope compositions (cf. acknowledgements). The Mochlos ingot is the only object from which a new sample was taken.

**Table 3:** Tin ingots analysed in the present study and in previous projects.

Site	Museum	Museum no.	Lab. no. CEZA	Sample designation in former studies	Type of sample	Analysed by	TIA	LIA	CC	M
Mochlos	AMA	None	MA-145558	None	Massive sample, corrosion	None	–	–	–	–
Haifa	NMM	8251	MA-175618	8251	Drillings, metallic	[21]	–	–	+	+
				8251		[52]	–	–	+	–
	NMM	8252	MA-175619	8252	Drillings, metallic	[21]	–	–	+	–
				8252		[52]	+	–	–	–
				G21		[32]*	–	–	+	–
				G21		[33]*	+	–	–	–
Hishuley Carmel	NMM	53/95 (95/2)	MA-175620	9; T9 or T14?	Drillings, metallic	[26]	–	+	–	–
	NMM	53/95 (95/3)	MA-175621	10; T6	Drillings, metallic	[26]	–	+	–	–
	NMM	82–132	MA-175668	1111/1A+B	Drillings, metallic	[24]	–	–	+	–
				HC1111/1		[31]	+	–	+	–
				HC1111/1		[16]	–	+	–	–
			(FG-883197)	1111/1; HDM 3231		[62]	–	+	+	–
				4; T1		[26]	–	+	+	–
	NMM	82–131	MA-175669	1111/2A+B	Drillings, metallic	[24]	–	–	+	–
				HC1111/2		[31]	+	–	+	–
				HC1111/2		[16]	–	+	–	–
			(FG-883198)	1111/2; HDM 3232		[62]	–	+	+	–
				7; T2		[26]	–	+	+	–
	NMM	82–130	MA-175670	1111/3A+B	Drillings, metallic	[24]	–	–	+	–
				HC1111/3		[16]	–	+	–	–
			(FG-883199)	1111/3; HDM 3233		[62]	–	+	+	–
				6; T3		[26]	–	+	+	–
	NMM	82–133	MA-175671	1111/4A+B	Drillings, metallic	[24]	–	–	+	–
				HC1111/4		[16]	–	+	–	–
			(FG-883200)	1111/4; HDM 3234		[62]	–	+	+	–
				1; T4		[26]	–	+	+	–
	NMM	None	MA-175672	1111/5A+B	Drillings, metallic	[24]	–	–	+	–
				HC1111/5		[16]	–	+	–	–
			(FG-883201)	1111/5; HDM 3235		[62]	–	+	+	–
				5; T5		[26]	–	+	+	–
	NMM	133-ד (53-ס)	MA-175673	2; T7	Drillings, metallic	[26]	–	+	–	–
	NMM	None	MA-175674	G8	Drillings, metallic	[32]	+	–	–	–
				G8		[73]	+	–	–	–
				G8		[33]	+	–	–	–
				8; T8		[26]	–	+	+	–
	NMM	314-ד (35/6-ס); 91–479	MA-175675	G13	Drillings, metallic	[32]	+	–	–	–
				G13		[73]	+	–	–	–
				G13		[33]	+	–	–	–
				13; T10		[26]	–	+	+	–
	NMM	26/92 (33/2)	MA-175676	14; T11	Drillings, metallic	[26]	–	+	–	–
	NMM	1637/89 (30/2)	MA-175677	12; T12	Drillings, metallic	[26]	–	+	–	–
	NMM	314-ד (35/36-ס); 1637/89 (30/3)	MA-175678	G15	Drillings, metallic	[32]	+	–	–	–
				G15		[73]	+	–	–	–
				G15		[33]	+	–	–	–
				15; T13		[26]	–	+	+	–
	NMM	26/92 (35/1)	MA-175679	G11	Drillings, metallic	[32]	+	–	–	–
				G11		[73]	+	–	–	–
				G11		[33]	+	–	–	–
				11, T9 or T14?		[26]	–	+	–	–
Kfar Samir south	NMM	81–609	FG-883202	81–609; HDM 3236	Drillings, metallic	[62]	–	+	+	–
	NMM	81–608	FG-883204	81–608; HDM 3238	Drillings, metallic	[62]	–	+	+	–
				81/608-5		[31]	+	–	–	–
	NMM	81–605	FG-883205	81–605; HDM 3239	Drillings, metallic	[62]	–	+	+	–
	NMM	81–604	FG-883206	81–604; HDM 3240	Drillings, metallic	[62]	–	+	+	–
	NMM	None	MA-176924	G19	Drillings, metallic	None	–	–	–	–
	NMM	None	MA-176925	G21	Drillings, metallic	[32]	+	–	–	–
	NMM	None	MA-176926	G22	Drillings, metallic	None	–	–	–	–
Uluburun	MUA	KW 197	FG-883208	KW 197	Massive sample, corrosion with some residual tin	[5]	–	–	+	–
				KW 197		[16]	–	+	–	–
				KW 197; HDM 3242		[62]	–	+	+	–
	MUA	KW 199	FG-883209	KW 199	Massive sample, corrosion	[5]	–	–	+	–
				KW 199; HDM 3243		[62]	–	+	+	–
	MUA	KW 203	FG-883210	KW 203	Fragments, corrosion from ingot core	[5]	–	–	+	–
				KW 203; HDM 3244		[62]	+	+	+	–
				KW 203; TR-35/155		[99]	–	–	+	–
	MUA	KW 203	FG-883211	KW 203A; HDM 3245	Fragments, corrosion from surface of ingot	[62]	–	+	+	–

The fourth column specifies the lab nos. of the actual samples used in this study, whereas the fifth column specifies the ID of the same or duplicate samples of the ingots from former investigations as compiled in the seventh column. All samples are permanently stored in the CEZA sample collection (Mannheim, Germany) and are publicly available. AMA, Archaeological Museum, Agios Nikolaos, Greece; NMM, The National Maritime Museum, Haifa; MUA, Bodrum Museum of Underwater Archaeology, Turkey; TIA, tin isotope analysis; LIA, lead isotope analysis; CA, chemical analysis; M, metallography; +, analysis was carried out;–, analysis was not performed. The find location of the samples tagged with an asterisk (*) was mistaken as ‘Atlit’ in the respective literature.

The Mochlos sample was embedded in epoxy resin for metallographic examination on polished section. It was ground with SiC papers up to 1200 grit and polished with diamond and alumina suspensions down to 0.25 μm. The microstructure was studied using optical (OM; Axioskop 40, Zeiss) and scanning electron microscopy (SEM; Evo MA 25, Zeiss). Analyses with an energy dispersive X-ray micro-analyser (EDX; Quantax 400, Bruker AXS) integrated in the SEM were carried out standardless to identify metallic and non-metallic phases and to estimate the bulk chemical composition of the ingot. In addition, the bulk composition (^55^Mn, ^57^Fe, ^59^Co, ^60^Ni, ^63^Cu, ^66^Zn, ^75^As, ^93^Nb, ^107^Ag, ^111^Cd, ^113^In, ^121^Sb, ^126^Te, ^181^Ta, ^182^W, ^197^Au, ^206^Pb, ^209^Bi) was determined directly on the polished cross-section using a laser ablation quadrupole inductively coupled plasma mass spectrometry approach (LA-Q-ICP-MS; ATL ArF 193nm, Resonetics and XSeries II Thermo Scientific). The badly corroded Uluburun objects were treated the same way, but only one of them was prepared metallographically (FG-883208). The other strongly corroded powder samples from the Uluburun tin ingots were analysed using pressed binderless pellets. Transient signals were recorded using Thermo PlasmaLab software. Signals were gas-blank-subtracted and spikes were excluded. The NIST 610 glass was used as external standard for quantification with ^122^Sn from the above-mentioned approach (EDX) as internal standard. An in-house excel-spread sheet was used for data-processing.

The chemical composition of the Israeli ingots was determined with the same quadrupole device, but using sample solutions. For this purpose, metal drillings were mechanically cleaned to remove surface contaminations. All samples (2–10 mg) were dissolved in a mixture of 6N HCl with small amounts of H_2_O_2_ in Teflon beakers on a hotplate (80°C). Thereafter, aliquots of the samples were diluted to 0.5N HCl and scandium, tamarium and rhenium (all Merck KGaA, Darmstadt, Germany) were added as internal standards. In case of solution measurements, tin concentrations are based upon 100% normalisation. A tin-lead metal standard (NF-2, Alpha Analytical Laboratories, Jersey City, USA) used for quality control mostly obtained results in good agreement with the reported values (Au, Bi, Sn_calc_ <5%; As, Cu, Sb, In within 5–10%; Cd, Ag 10–20%). Iron (20%), zinc (400%) and nickel (90%) values are reported as strongly influenced by segregation effects and were therefore not reliable for quality control.

Aliquots of the sample solutions were used for tin isotope analysis (TIA) after dilution with deionised water and 0.4 N HNO_3_ + concentrated HF and processed further as described in detail by Brügmann et al. [74]. No chemical separation was necessary before the isotopic measurements because the metal consisted of almost pure tin, and potential isobaric interferences of cadmium, antimony, arsenic or tellurium were not observed. An antimony solution with known isotope composition (Specpure ICP–AES, Lot#. PSBH24/13, supplied by Fisher Chemicals) was added to the sample solutions as an internal standard in order to correct the mass discrimination occurring during the measurements in the mass spectrometer.

A Neptune Plus (Thermo Scientific, Bremen, Germany) multi-collector mass spectrometer with inductively-coupled plasma ionisation (MC-ICP-MS) was employed for the isotopic analyses. It was equipped with nine Faraday cups measuring simultaneously seven stable tin isotopes (^116^Sn, ^117^Sn, ^118^Sn, ^119^Sn, ^120^Sn, ^122^Sn, ^124^Sn) and two antimony isotopes (^121^Sb, ^123^Sb) for mass bias correction. Since there is still no internationally certified tin reference material, an in-house standard was prepared from ultraclean tin metal (Puratronic, Batch W14222, Johnson Matthey, Royston, GB) by dissolving it in HCl. This metal had already been used in previous studies [31–33; 64; 70; 72; 75–81]. The isotopic ratios reported here are related to the in-house standard and are given in the delta notation in units of permil (‰) with ^120^Sn as the common denominator. δ^124^Sn, which is used for discussion hereafter, would thus represent δ^124^Sn/^120^Sn [74]. For better comparisons with other studies the isotope compositions are also given as δSn in in ‰ per atomic mass unit (‰ u^-1^) in the supplementary material (S2 Table).

**Fig 5:**
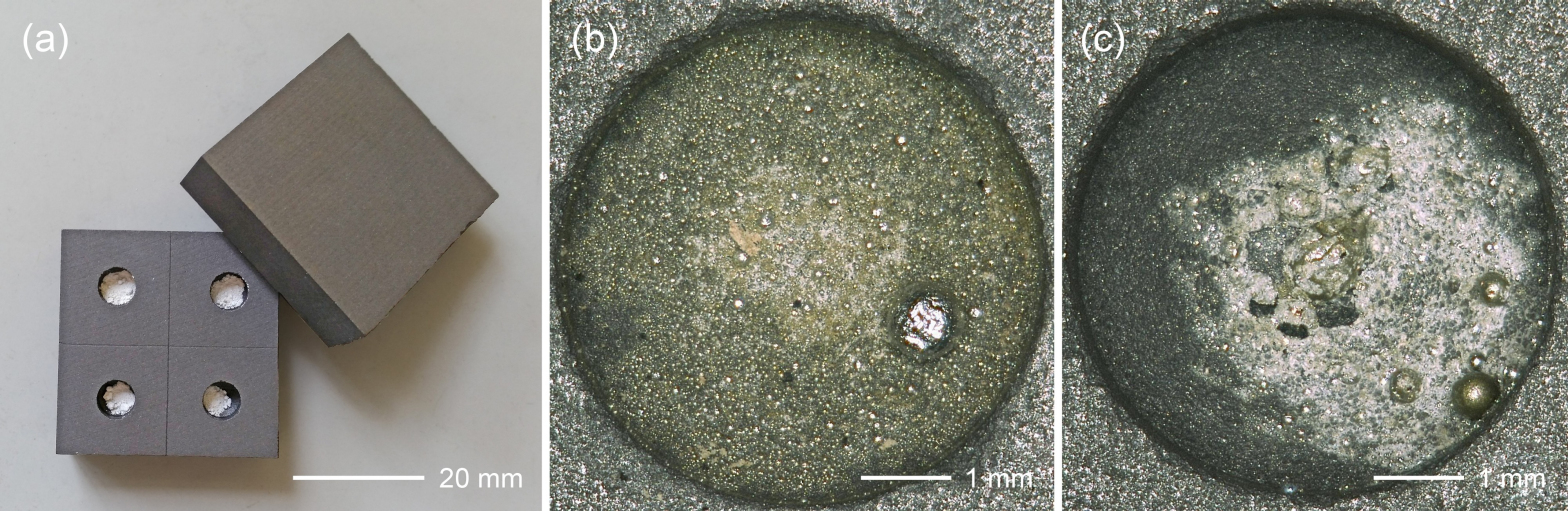
**Graphite plates used for the reduction of the corroded tin samples (a).** Resulting tin beads from the white crust, sample MA-145558a (b) and reduced tin from the heterogeneous core, sample MA-145558b (c) of the Mochlos tin ingot (photographs: D. Berger).

In contrast to the metallic samples, the corroded specimens of the Mochlos and Uluburun ingots had to be converted to tin metal prior to TIA because common corrosion products of tin, such as stannic oxide (SnO_2_) and hydrated stannic oxide (SnO_2_·nH_2_O) [82–83], are almost insoluble in acids (although stannic oxide is identical with cassiterite we use the term for the corrosion product in order to distinguish it from the natural ore mineral). Conversion to tin metal was achieved by reduction of a small amount of pulverised material (~10 mg) in a muffle furnace at 950°C, according to the protocol established by Berger et al. [70]. In order to prevent tin loss during heating due to the formation of volatile SnO [84], reduction was performed in presence of potassium cyanide (KCN) using graphite crucibles (Fig 5). This is the most reliable procedure for cassiterite/stannic oxide reduction, as no tin loss and isotopic fractionation due to evaporation has been observed so far [64; 70; 85]. After reduction, the tin metal was processed for TIA like the Israeli specimens. The approach was the same for the preparation and characterisation of Eurasian cassiterite ores that are employed for comparison in this study. Ore samples are summarised in S3 Table, but without specification of numerical values of their tin isotope composition. Values and geological interpretation will be supplied in a forthcoming PhD thesis (J. Marahrens). The combined analytical uncertainty (2SD) for multiple measurements of certified bronze reference materials (BAM 211, IARM-91D) arising from the sample processing and the measurements was ± 0.02 ‰ for δ^124^Sn [74]. All analytical errors specified for the tin isotope ratios of the individual tin samples are given as 2SD.

The lead isotope ratios of most of the tin ingots were also determined using solution aliquots and an established and improved analytical protocol [86] with substantially improved precision of better than 0.003% in all ratios. The measurements were also performed in the Mannheim laboratory with the Neptune Plus mass spectrometer.

In addition to the chemical and isotopic investigations, X-ray diffraction analysis served for the determination of the mineralogical composition of the corroded ingot samples. Powder samples of the Mochlos and Uluburun ingots, which were prepared for TIA, were analysed in the Institute for Geosciences, Heidelberg University (H.-P. Meyer), Germany, with a D8 ADVANCE eco powder diffractometer (Bruker AXS GmbH, Karlsruhe, Germany). Analytical parameters are documented in S1 Table along with the other analytical instruments used in this study.

## 4. Results and discussion

### 4.1. Microstructure of the Mochlos ingot

**Fig 6:**
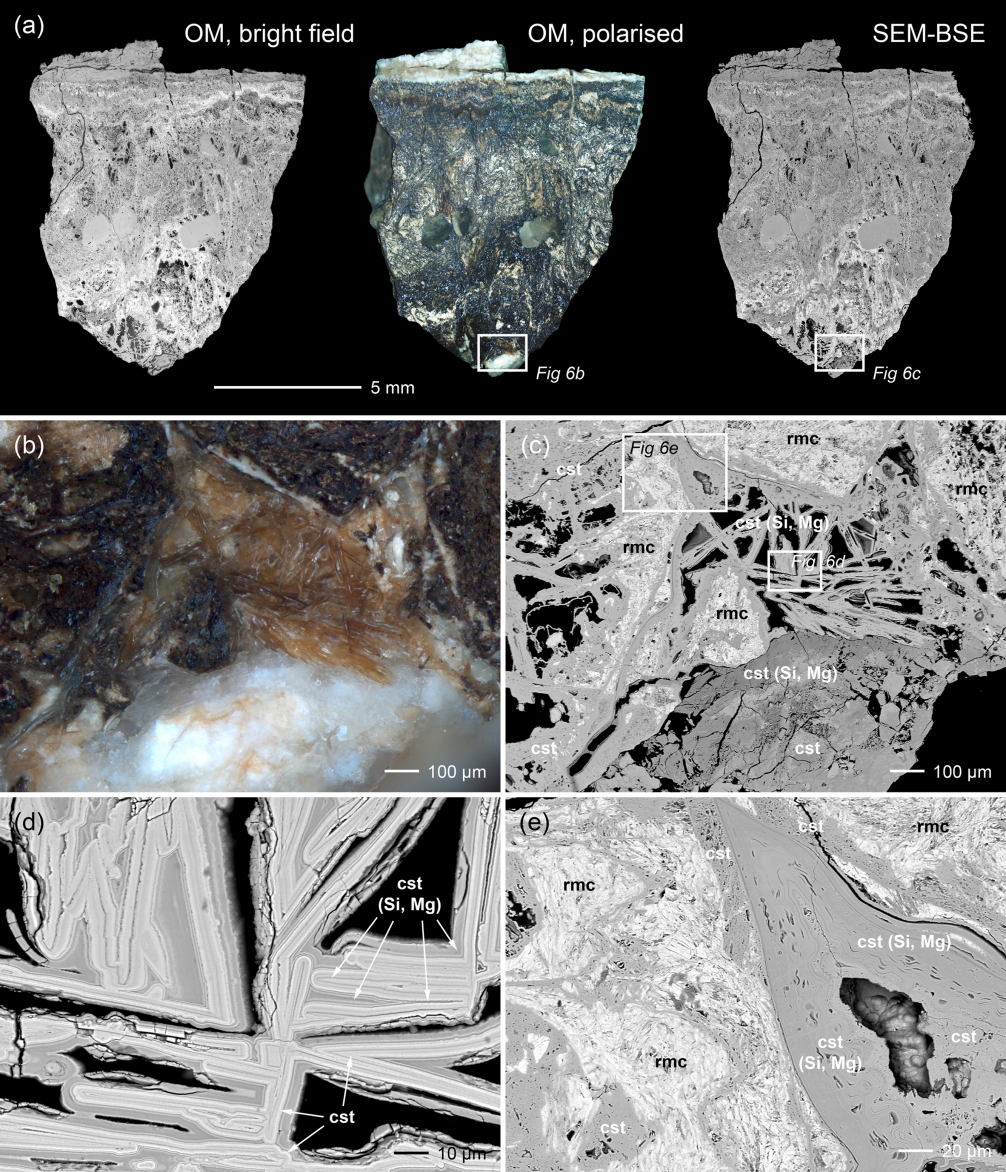
Microscopic documentation of the corroded ingot sample from Mochlos. (a) overview images from optical microscopy (bright field illumination on the left and polarised light in the middle) and SEM (backscattered electron image on the right); (b) detail from optical microscopy (polarised light) of the area specified in (a) showing differently coloured corrosion; (c) SEM-BSE image of the same area as in (b) with identified mineralogical phases; (d) and (e)–selected areas of (c) seen with higher magnification in which the phases romarchite (rmc), stannic oxide (cst) and silicon and magnesium containing stannic oxide (cst (Si, Mg)) are specified (images: D. Berger).

**Fig 7:**
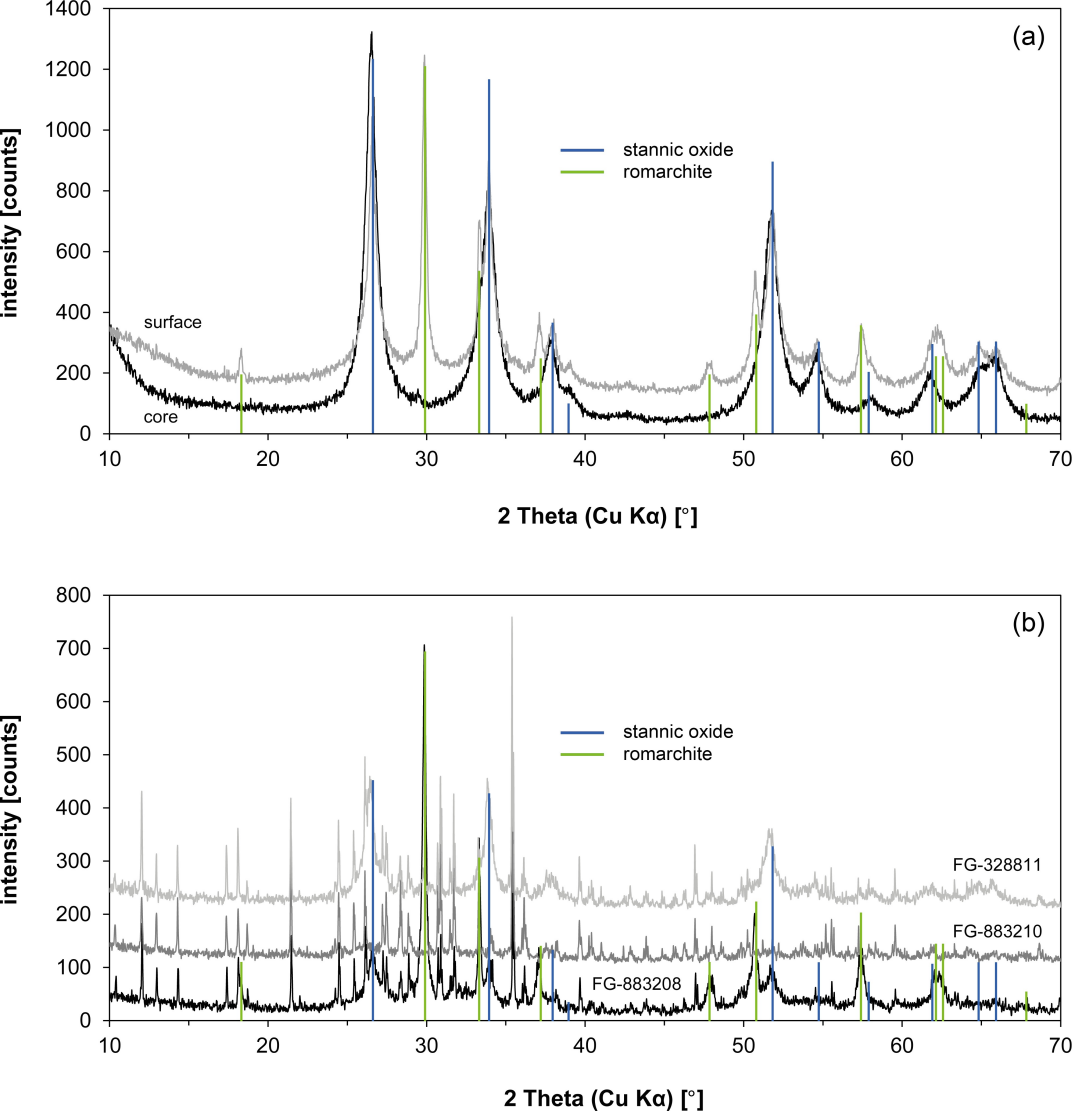
Results of X-ray diffraction analysis carried out on the corroded ingots of Mochlos and Uluburun. The comparison of the surface and the core of the Mochlos tin reveals a mixture of stannic oxide and romarchite in the interior and almost pure stannic oxide at the surface (a). No reflexes of grey tin are actually observed (most intense peak at 23.701°). The Uluburun ingots (b) additionally contain abhurite or are completely composed of this mineralogical phase (all peaks that are not specified belong to abhurite). No grey tin is present (diagrams: D. Berger; data: H.-P. Meyer, University Heidelberg).

Fig 6 shows the microstructure of the Mochlos tin ingot and reveals its severely corroded condition. The former tin metal had been completely converted to several corrosion products that can be distinguished by OM due to their different colours and reflection properties. The examination with the SEM (backscattered mode) shows different shades of grey, depending on the mean atomic numbers of the compounds (Fig 6). The figures and the chemical analyses indicate that the surface is covered by a homogenous and dense layer of whitish stannic oxide (*cf*. Table 4, point no. 1), while the interior of the ingot exhibits a more porous structure with an alternating sequence of transparent, white, brown to almost black corrosion products. Analyses with EDX and XRD show stannic oxide to be the predominant corrosion product in the interior as well, but some hydroromarchite (Sn_3_O_2_(OH)_2_) and a large fraction of romarchite (SnO) could also be identified (Table 1 and Figs 6C and 6E and 7A). No grey tin (‘tin pest’), which previously was thought to be the predominant phase in the ingot [6–7], is present, and this is also true for the corroded ingots from Uluburun (Fig 7B). The disintegration of the tin from Mochlos to a pile of gravel and powder is thus just the consequence of severe corrosion (cf. Fig 3).

**Table 4:** Chemical composition (semi-quantitative) of corrosion products of the Mochlos tin ingot determined with SEM-EDX at various positions of the polished cross-section (data: D. Berger).

Point no.	O	Mg	Si	S	Cl	Sn
*Stannic oxide*						
Point 1	21.5					78
Point 2	21.6			0.16	0.14	78
Point 3	21.2				0.07	79
Point 4	21.3					79
Point 5	21.3					79
**mean**	**21.3**			**0.16**	**0.10**	**79**
*Silicon- and magnesium-rich stannic oxide*						
Point 21	21.9	0.24	0.22			78
Point 22	21.6	0.51	0.42			77
Point 25	21.2	0.34	0.45			78
Point 26	21.5	0.25	0.13		0.10	78
Point 42	21.2	0.54	0.60			78
**mean**	**21.5**	**0.38**	**0.36**		**0.10**	**78**
*Romarchite*						
Point 18	11.5				0.47	89
Point 20	11.1				0.19	88
Point 23	11.4					88
Point 28	12.8				0.23	88
Point 29	13.1		0.08		0.46	89
**mean**	**12.0**		**0.08**		0.19	89
*Hydroromarchite*						
Point 45	16.4				0.15	83
Point 46	17.3				0.22	82
**mean**	**16.9**				**0.18**	**83**

Romarchite is black, while stannic oxide is known to adopt a wide range of colours [87]. Previous studies on the optical properties of geologically formed SnO_2_ mainly identified impurities (micro-inclusions, foreign ions) being responsible for the crystal colours [88–90]; however, structural defects and growth mechanisms were also suggested [91–92]. This problem on the origin of the colour is not yet fully understood, even for stannic oxide on archaeological tin objects.

On closer inspection of the sample, SnO_2_ crystals of different colours can be observed side by side Fig 6B). These crystals have different magnesium, silicon and chlorine contents; however, no overall correlation between crystal colour and chemical composition could be established (Table 4). Compact layers and porous masses of impurity-free oxide appear as transparent or as white as stannic oxide that contains the above-listed light elements (*cf*. Fig 6B and 6C). The alteration products exhibit brownish colours only in banded structures, which seem to reflect alternating layers of impure and pure SnO_2_ (Fig 6D and 6E). This is the case, for instance, for stannic oxide with well-developed tabular crystals up to 500 μm in length that are observed within pores in several places in the core of the sample (Fig 6D). The banded growth of these crystals indicates the dissolution of metallic tin or primary tin corrosion phases (romarchite, hydroromarchite) and the subsequent re-precipitation of stannic oxide as the final corrosion product. According to these observations and to the study of Dunkle et al. [83], this texture most likely formed when romarchite and hydroromarchite dissolved on progressive corrosion and transformed into SnO_2_. The mechanism of coupled dissolution-re-precipitation is well known to occur in metamorphic, hydrothermal and alteration events during geological processes and is suggested to be the principal process here as well [93]. In the course of this process, the former compact tin metal was transformed into a highly porous matrix with angular pores. This porosity hence differs from that produced during the casting of a tin melt (rounded pores) as shown by examinations of archaeological tin and tin alloys [83; 94].

### 4.2. Chemical composition of the tin ingots

The use of trace element concentrations for provenance studies of ancient tin requires several preconditions: 1. Known chemical composition of tin ores; 2. Known behaviour of the trace elements during tin ore smelting, and 3. Determination of the chemical composition of tin metal with sensitive analytical methods. Unfortunately, data are rarely available for evaluating all three aspects, which complicates the provenancing of archaeological tin objects. The paucity of tin ore data makes it especially difficult to decide which elements are useful for fingerprinting and which are not. However, smelting experiments [63; 79; 95–96] show that only a few minor and trace elements in tin ores might be diagnostic since many do not partition quantitatively into the metal during the smelting process and either get lost to the slag or are considerably depleted in the metal. Promising elements are antimony, silver, selenium, indium, tellurium, mercury and gold and possibly the rare earth elements lanthanum and europium. To a lesser degree iron, tungsten, tantalum, scandium and hafnium could be useful [95–97]. As stated by Grant [96], trace elements alone are not unambiguous tracers for tin sourcing though. Nevertheless, the chemical composition of the tin ingots determined in this study can be used to compare and eventually to distinguish the objects. Moreover, the data can be related to the results of the recent publication of Wang et al. [94] which studied MBA tin ingots (1300–1150 BCE) from sites off the coast near Salcombe and the Erme Estuary, Devon, United Kingdom (Fig 1). There is also data available for LBA artefacts and for 32 ingots from the Uluburun wreck [45; 98–99]. However, the data of the latter is of limited use because of a differing element selection and analytical method (ICP-OES).

The chemical analyses of the Israeli ingots (Table 5) confirm the results of previous investigations of some of the objects (cf. Table 3) in that they reveal unalloyed tin with low levels of impurities. This finding excludes stannite (Cu_2_FeSnS_4_) and other tin containing ores such as secondary tin oxyhydrates of the Mushistonite type ((Cu,Zn,Fe)Sn(OH)_6_), because higher concentrations of impurity elements like iron, copper, zinc and lead would be expected.

**Table 5:** Bulk chemical composition of the tin ingots determined with Q-ICP-MS and LA-Q-ICP-MS (for the Mochlos and Uluburun ingots).

Site	Lab. no. (CEZA)	Mn	Fe	Co	Ni	Cu	Zn	As	Nb	Ag	In	Sn	Sb	Te	Ta	W	Au	Pb	Bi
Mochlos	MA-145558a*	0.3	9	15	93	29	0.5	13	<0.26	<1.1	17	99.98	15	*1*.*9*	<0.12	<0.03	<0.03	1.9	6
	MA-145558b*	<0.7	<1.8	12	77	53	4	8	<0.26	<1.1	34	99.98	14	*1*.*9*	<0.12	<0.03	<0.03	5	7
Haifa	MA-175618	7	402	<15	<16	<6.5	*64*	<196	<0.6	<1	30	99.94	20	*16*	<1	<0.2	2.2	11	13
	MA-175619	9	857	<15	<16	<6.5	*95*	<196	<0.6	<1	29	99.89	19	*15*	<1	<0.2	1.4	10	16
Hishuley Carmel	MA-175620	10	1348	<15	<16	<6.5	*8*	<196	1.4	<1	29	99.85	17	*17*	<1	*2*.*4*	1.2	13	20
	MA-175621	12	432	<15	174	<6.5	*94*	*221*	1.4	<1	23	99.90	15	*21*	<1	*2*.*0*	1.2	21	12
	MA-175668	14	312	<15	<16	<6.5	*76*	*305*	1.0	<1	27	99.92	15	*18*	<1	*1*.*8*	2.0	22	31
	MA-175669	14	1358	<15	<16	<6.5	*35*	*351*	0.8	<1	26	99.80	105	*18*	<1	*1*.*2*	0.8	46	34
	MA-175670	17	893	<15	<16	<6.5	*96*	*442*	0.8	<1	26	99.85	15	*18*	<1	*1*.*2*	0.8	22	4
	MA-175671	18	<96	<15	51	20	*70*	463	1.8	19	<1.6	99.92	4	*18*	<1	*2*.*4*	135	15	2
	MA-175672	21	<96	<15	22	11	*85*	<196	<0.6	<1	30	99.98	18	*16*	<1	*1*.*4*	0.6	13	8
	MA-175673	20	476	<15	<16	<6.5	*37*	<196	<0.6	<1	67	99.93	19	*18*	<1	*1*.*4*	<0.3	6	14
	MA-175674	25	392	<15	<16	<6.5	*51*	<196	<0.6	<1	36	99.91	332	*18*	<1	*2*.*4*	0.8	26	4
	MA-175675	27	<96	<15	<16	<6.5	*467*	<196	<0.6	<1	24	99.94	11	*17*	<1	*0*.*8*	0.4	12	12
	MA-175676	27	<96	<15	<16	<6.5	*54*	<196	<0.6	<1	55	99.98	18	*19*	<1	*0*.*6*	0.8	11	10
	MA-175677	28	<96	<15	<16	<6.5	*46*	<196	<0.6	<1	48	99.95	385	*17*	<1	*0*.*6*	1.4	20	3
	MA-175678	28	<96	<15	<16	<6.5	*56*	<196	<0.6	<1	56	99.98	20	*18*	<1	*0*.*6*	0.6	10	56
	MA-175679	30	<96	<15	<16	<6.5	*37*	<196	<0.6	<1	58	99.98	14	*17*	<1	*0*.*6*	<0.3	6	15
Kfar Samir south	FG-883202	n. a.	n. a.	n. a.	n. a.	n. a.	n. a.	n. a.	n. a.	n. a.	n. a.	n. a.	n. a.	n. a.	n. a.	n. a.	n. a.	n. a.	n. a.
	FG-883204	<1.3	<96	<15	<16	<6.5	<4.7	<196	<0.6	<1	21	99.99	13	<1.3	<1	*8*	<0.3	19	18
	FG-883205	8	268	<15	<16	15	<4.7	<196	<0.6	<1	24	99.96	14	<1.3	<1	*13*	<0.3	55	16
	FG-883206	14	410	<15	53	32	<4.7	<196	<0.6	<1	34	99.88	45	<1.3	<1	*9*	<0.3	547	60
	MA-176924	<1.3	<96	<15	<16	19	16	<196	<0.6	<1	56	99.99	14	<1.3	<1	<0.2	<0.3	2	7
	MA-176925	<1.3	<96	<15	43	40	53	<196	<0.6	<1	53	99.98	12	<1.3	<1	<0.2	<0.3	8	4
	MA-176926	<1.3	<96	<15	<16	27	<4.7	<196	<0.6	<1	20	99.99	12	<1.3	<1	<0.2	<0.3	9	7
Uluburun	FG-883208*	<0.7	30	*15*	*92*	26	0.32	29	0.26	2	3	99.95	29	*3*	<0.12	<0.03	0.58	42	186
	FG-883209*	9	125	*16*	*96*	370	173	73	0.8	9	2	99.73	35	*3*	0.9	0.59	7	70	230
	FG-883210	n. a.	n. a.	n. a.	n. a.	n. a.	n. a.	n. a.	n. a.	n. a.	n. a.	n. a.	n. a.	n. a.	n. a.	n. a.	n. a.	n. a.	n. a.
	FG-883211*	74	813	*17*	*121*	559	49	35	2	106	7	99.15	18	*2*	<0.12	<0.03	47	62	671

All values are reported in μg g^-1^ except for Sn which is given in mass%. Note: Samples tagged with an asterisk (*) were analysed with LA-Q-ICP-MS and data is mean values of two to eight single measurements. All other samples were measured using solution Q-ICP-MS. Values given in italic numbers suffered interferences during analysis, so values should rather be taken as semiquantitative. ‘n. a.’ means ‘not analysed’ (data: N. Lockhoff).

**Fig 8:**
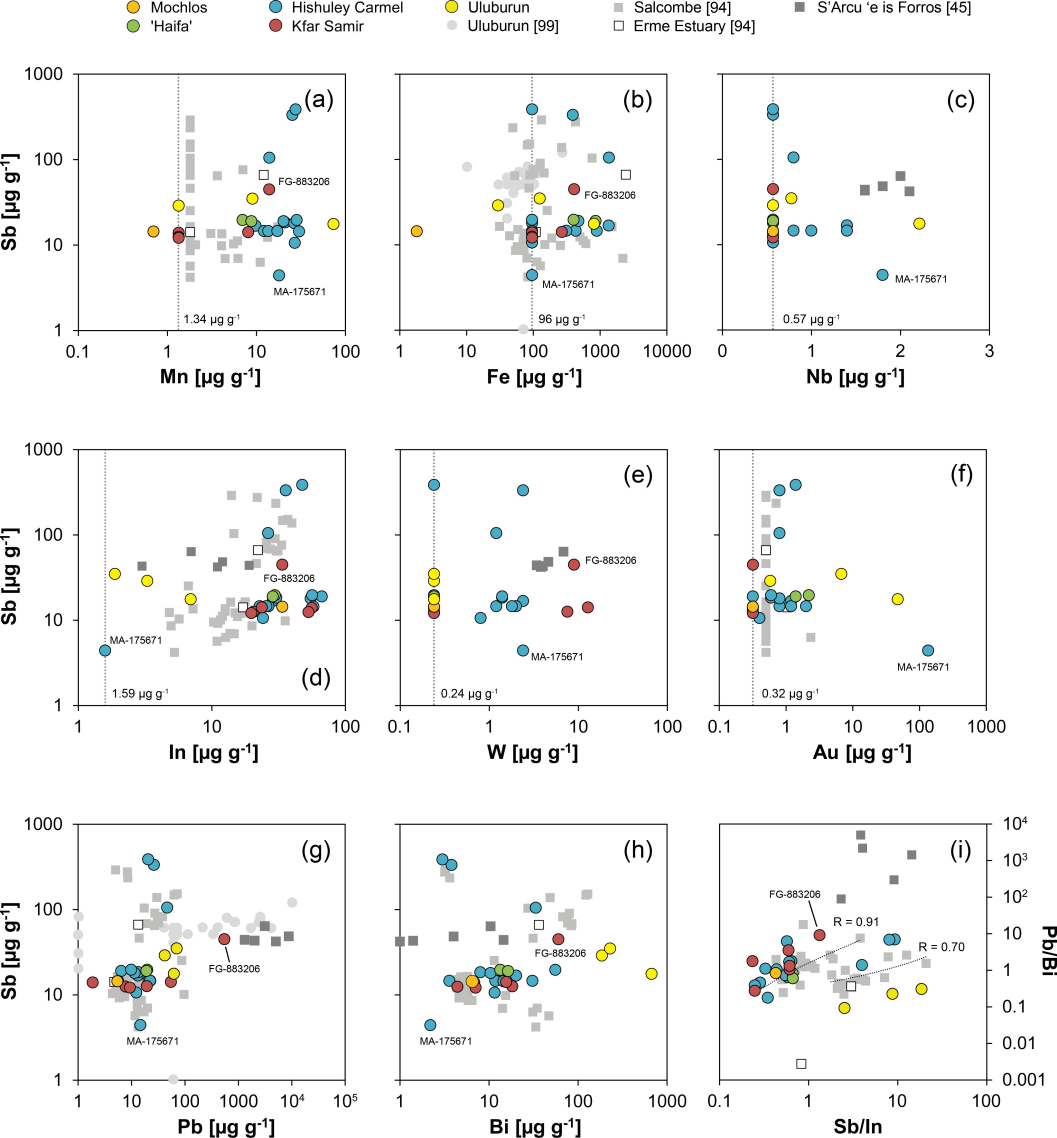
Trace element composition (vs. antimony) of ingots examined in this study compared with presumed MBA and LBA tin ingots and objects from Salcombe, the Erme Estuary, Uluburun and S’Arcu e is Forros, Sardinia. The vertical dotted lines and the numbers represent the detection limits of the Q-ICP-MS for the respective element, values for Mochlos and Uluburun are often lower due to the use of LA-Q-ICP-MS. The correlation coefficients R in (i) were derived from the two groups from the Salcombe tin ingots and serve for comparative purposes only (MA-175671 not shown in this diagram). Legend applies to all diagrams (diagrams: D. Berger, data: L. Lockhoff; [45; 94; 99]).

The highest concentrations of all impurities were determined for iron with up to 1350 μg g^-1^ (Fig 8B). Strikingly, half of the Hishuley Carmel ingots exhibit high iron content whereas iron of the other half is below the detection limit of the analytical method (96 μg g^-1^). This observation could be attributed to contamination by corroded material, but since the samples were cleaned prior to analysis it is more likely that it reflects differences in the reduction process (e.g. temperature, oxygen content of the atmosphere) of the tin ores or the intensity of refining after smelting [98]. It is of interest to note that the ingots from Haifa show the same elevated level of iron that could indicate a similar less-refined condition like one part of the Hishuley Carmel assemblage.

Matches between the Haifa and Hishuley Carmel ingots are also observed for other trace elements, in particular for antimony, lead, bismuth and indium (Fig 8). Antimony was detected with up to 385 μg g^-1^, but the majority of the ingots have antimony concentrations between 10 and 20 μg g^-1^. Lead and bismuth levels are generally low with values ranging from 6 to 46 μg g^-1^ and 2 to 56 μg g^-1^, respectively. Indium is present in concentrations of 23 to 58 μg g^-1^, and in the bivariate diagram with antimony four groups become discernible in the Hishuley Carmel ingots following at least two trends (Fig 8D). The Haifa ingots can be assigned to one of these groups with medium high contents of both indium and antimony. Such a grouping, however, is not revealed in diagrams with the other elements.

Copper, silver and tellurium concentrations are low throughout the Haifa and the Hishuley Carmel objects and often below the detection limits of our ICP-MS measurements (Table 5 and Fig 8). Tantalum as a possible diagnostic element [95] was not detected in any sample, whereas niobium could be observed in some, and tungsten in all of the Hishuley Carmel tin ingots (Fig 8C and 8E). The Haifa ingots did not contain measurable concentrations of the latter three elements, but it is questionable that these elements could represent fingerprints of the tin source. It is more likely that they merely reflect the conditions during tin ore smelting because they usually behave lithophile and thus partition to the slag. Depending on the redox conditions during the smelting process and the valance state in minerals (accompanying cassiterite), tungsten and niobium can, however, be transferred to a limited extent to the metallic phase [100]. The same applies to manganese that is normally not reduced during smelting. Nevertheless, in the ingots of both assemblages it is present with concentrations between 7 and 30 μg g^-1^, and surprisingly a positive correlation between indium and manganese is observed for the Hishuley Carmel items (R = 0.76, n = 14).

**Fig 9:**
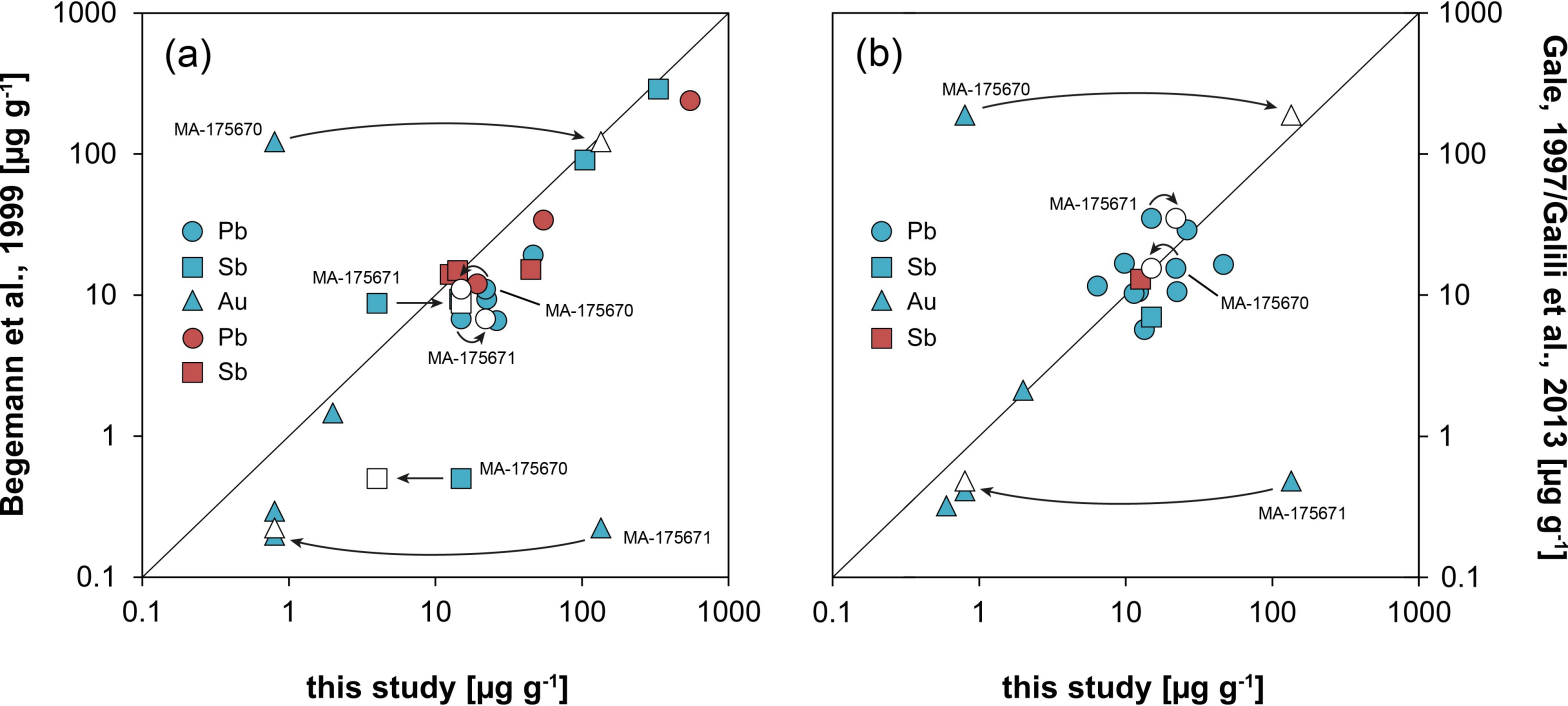
Chemical data of the Hishuley Carmel (blue symbols) and Kfar Samir (red symbols) tin ingots collected in this study. They are compared with data of the same objects from previous studies of Begemann et al. [62] (a) and Gale [31]/Galili et al. [26] (b). The arrows indicate interchangement of data of the respective samples after the recognition of sample confusion (diagrams: D. Berger).

Gold was also detected in the tin from both sites with 2.2 μg g^-1^ at maximum (Fig 8E). However, ingot no. MA-175671 (= HC1111/4; T4) is different from all other tin ingots from Israel since it has a very high gold (135 μg g^-1^) and the lowest indium (<1.6 μg g^-1^) and antimony (4 μg g^-1^) concentrations besides detectable silver (19 μg g^-1^) and copper (20 μg g^-1^). Comparable values were observed by Begemann et al. [62] and Stos-Gale et al. [16])/Galili et al. [26] for ingot T3, after having examined two separate samples of the same object with neutron activation analysis ([62]: FG-883199 = 1111/3; [16]/[26]: HC1111/3 = T3 = MA-175670; cf. Table 3). Because we analysed Stos-Gale et al.’s sample (MA-175670) and got results matching their sample HC1111/4 (T4), we fear sample confusion in this case due to the blatant coincidence with our data of ingot T4 (T3 ↔ T4) (This problem will be addressed again in the lead isotope section). Finally, we cannot reconstruct when the potential confusion might have occurred, but irrespective of this issue, the data of MA-175670/MA-15671 (after interchanging of the data sets) and the other Hishuley Carmel ingots show a reasonable agreement for lead, antimony and gold with the data of the other research groups (Fig 9). Large discrepancies were only observable for iron, cobalt, nickel, zinc and arsenic. The latter four elements in our data sets presumably had large blanks or interferences (therefore, this data is not discussed in more detail), and iron could be inhomogenously distributed in the tin drillings (for Fe cf. [98]). In any case, the composition of MA-175671 suggests that the ingot T3 was smelted from another tin ore concentrate of possibly another tin source than the remaining Hishuley Carmel ingots. The elevated gold and silver contents could be an indication for an alluvial source of the tin [101].

The elemental pattern of the two ingots from Haifa on its part suggests that they are identical in terms of chemistry. Thus, they could have been produced from the same tin ore and even from the same metal batch. Moreover, as already stated above, their gold, lead and bismuth concentrations agree well with all of the Hishuley Carmel ingots, and their antimony, indium and iron with most of them (Fig 8). Thus, the assumption of a common origin for both cargoes appears reasonable.

The Kfar Samir south ingots show a trace element pattern similar to that of the other Israeli ingots, especially regarding their indium (20–56 μg g^-1^), antimony (12–45 μg g^-1^), lead (2–55 μg g^-1^), bismuth (4–60 μg g^-1^) and silver (<1 μg g^-1^) concentrations (Table 5 and Fig 8). What seems to differentiate them from the other objects are their gold and copper contents, which were either below the detection limit (Au: <0.32 μg g^-1^) or higher than those of the ingots from Hishuley Carmel and Haifa (Cu: 19–40 μg g^-1^) (Fig 8C and Table 5). In addition, iron and manganese could be detected in just two samples (Fig 8A and 8B). If present, gold could indicate an alluvial tin source; however, copper and gold are also often associated with primary tin deposits. The lack of gold is thus not convincing evidence for the use of primary tin, albeit the high copper concentration could be a fingerprint. Since the concentrations of gold and other elements could vary over several orders of magnitude within single deposits, it is not certain that the small concentration differences between the tin ingots reported here can help to narrow down the origin of the tin ores. The same is true also for other elements, but the presence and concentrations of manganese, iron, lead, bismuth and tungsten characterise two groups within the ingots from the Kfar Samir wreck that could be interpreted metallurgically (Table 5). Thus, as with the different groups of the Hishuley Carmel ingots the distinct groups of Kfar Samir might indicate disparate states of refining or different numbers of re-melting events.

The chemical composition of the Mochlos ingot is also shown in Fig 8 (cf. Table 5). Because of its corroded nature the chemical data of the Mochlos tin, however, can only be used for rough comparison with the non-corroded tin items. In this respect, the data from the brownish coloured ingot core (MA-145558b) appears more meaningful than that from its whitish surface crust (MA-145558a) since the metal in the interior corroded later than the now whitish surface (Table 5). It could thus be less biased by impurities and depletion or enrichment processes as it was shielded by an initially formed oxide layer.

Provided no significant depletion or enrichment of elements occurred during the corrosion of the tin metal, the chemical composition of the Mochlos ingot agrees well with that of the Hishuley Carmel, Kfar Samir south and the Haifa ingots (cf. Fig 8). For example, their average indium and antimony concentrations (37 μg g^-1^ and 17 μg g^-1^, respectively) overlap with those determined in the core of the Mochlos ingot (34 and 14 μg g^-1^, respectively). Lead (5 μg g^-1^), bismuth (6 μg g^-1^), iron (<1.8 μg g^-1^) and manganese (<0.7 μg g^-1^) on their part tend to be at the low end of the concentration range, whereas the copper concentration is higher (53 μg g^-1^) than those in the other Mediterranean ingots (Table 5). A more detailed examination of the element distribution suggests that the chemical composition of the tin from Mochlos best matches the composition of the three ingots MA-176924 to MA-176926 from Kfar Samir. Gold, silver, niobium, tantalum and tungsten were below the detection limit; thus, these elements provide no information on the relationship between the ingots. Regarding the state of preservation, the low iron contents are surprising because iron is abundant in soil environments and is often incorporated in tin corrosion products [83; 92]. In addition, since no iron-tin intermetallic compounds (FeSn, FeSn_2_) were observed in the microstructure of the ingot (see above), the low iron suggests that either iron minerals were not reduced during smelting [102] or–more likely–that the tin underwent some kind of refining process which effectively removed the intermetallic components [94; 98].

**Fig 10:**
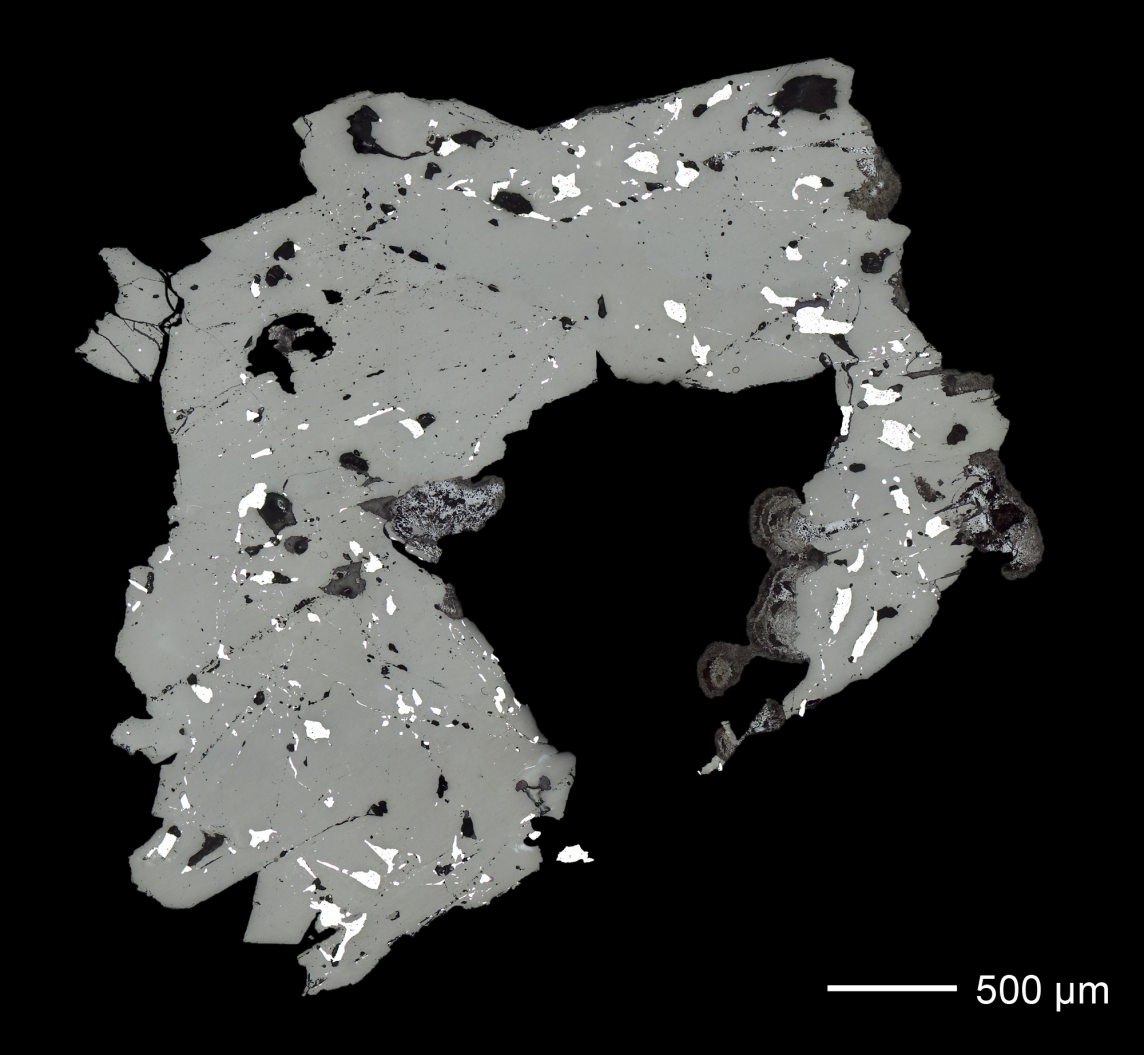
Cross-section of tin ingot KW 197 (FG-883208) from the Uluburun shipwreck showing residual tin metal embedded in a matrix of corrosion products (mainly abhurite). The glossy tin patches were examined with LA-Q-ICP-MS as reported in Table 5 (photo: D. Berger).

The chemical composition of the three Uluburun ingots included in this study is very different from the remaining tin objects. Although severely weathered, ingot KW 197 (FG-883208) fortunately contains some tiny patches of residual tin (Fig 10) that were analysed with LA-Q-ICP-MS at different positions. The residual tin metal is thus regarded to have a composition very close to that of the original tin ingot, and in this case the data can be used to evaluate the influence of the corrosion on the composition of the other two ingots with no metal leftovers (FG-883209, FG-883210). Table 5 and Fig 8 illustrate that many elements (Fe, Mn, Cu, Zn, Nb, Ag, Au, Bi) are enriched in the corroded items relative to the residual patches of tin metal in ingot KW 197 (FG-883208). Consequently, the concentrations given for FG-883208 can only be regarded as an upper limit of the original content of the respective elements. However, antimony, indium and lead do not show a marked enrichment and can be used for discussion. In this regard, the Uluburun ingots are richer in lead (42–70 μg g^-1^) and antimony (18–35 μg g^-1^), and significantly poorer in indium (2–7 μg g^-1^) compared with the ingots from Mochlos and Israel. In addition, ingot KW 197 has higher concentrations of silver (2 μg g^-1^) and bismuth (186 μg g^-1^). This data agrees well with the analyses (ICP-OES) carried out by Hauptmann and co-workers [99] on 32 Uluburun ingots, and although there is no data for all elements (especially for In and Bi) it is evident that the tin from Uluburun is very different from the other ingots. They have systematically higher copper, silver, antimony and lead concentrations (Fig 8G; [99]) which are more comparable to those of some LBA tin objects and tin ores found on Sardinia (Fig 8G; [45]). An origin differing from that of the Mochlos and Israeli ingots is therefore likely for the tin from Uluburun.

From the above description it can be assumed that lead, bismuth, antimony and primarily indium are important for sourcing the tin. The latter element occurs in tin deposits only in sulphidic assemblages, often incorporated in the crystal lattice of sphalerite, stannite or cassiterite or as micro-inclusion of indium minerals in chalcopyrite, sphalerite, stannite and cassiterite [103–104]. Hence, if indium is present in tin metal the parental tin ores should have contained indium-bearing sulphides and this suggests a polymetallic character [105–107]. If this is the case, many tin occurrences in central Asia and Egypt represent unlikely sources as their indium contents in cassiterite are often lower than 50 μg g^-1^ [97; 108]. European cassiterite mineralisations are rarely indium-rich as well [45; 103–104; 109–110], but a major exception seems to be the deposits in Cornwall/Devon, and especially those associated with the Carnmenellis and St. Agnes granites having cassiterites with high indium contents of more than 300 μg g^-1^ [105; 111]. Interestingly, the Salcombe ingots found offshore the Devon coast exhibit indium contents similar to those of the Mediterranean ingots (Fig 8D; [94]. If also antimony, lead and bismuth are considered in plotting a four-element diagram (Pb/Bi vs. Sb/In), many of the Israeli ingots and the piece from Mochlos match the British items (Fig 8I). The latter distribute along two discrete trends with correlation coefficients of R = 0.91 (n = 21) and 0.70 (n = 17), respectively, and the majority of the Israeli tin ingots follow one of these trends which is characterised by lower antimony contents (R = 0.69, n = 19). Since a tin source on the British mainland is obvious for the Salcombe ingots [94], this observation might provide additional evidence that most of the Israeli and Mochlos ingots were as well made from British tin ores. This conclusion does not *a priori* exclude those ingots from Hishuley Carmel that turned out to be different in their chemical composition (Fig 8I, right hand side). But, because they follow another trend, a different tin source from another region or deposit was certainly used for their production which is certainly true also for tin ingot MA-175671. With the chemical composition alone it is, however, not possible to decide whether this source was located in the British Isles or somewhere else, so additional information is needed.

### 4.3. Lead isotope systematics of the tin ingots

Cassiterite is characterised by very low concentrations of lead because the crystal lattice of SnO_2_ will not incorporate significant amounts of lead ions (Pb^2+^). On the contrary, the concentrations of uranium (U^4+^) in SnO_2_ are commonly much higher than that of lead [112–113] and the resulting U/Pb-ratios from the uranium decay lead to strongly radiogenic lead isotope ratios (^206^Pb/^204^Pb, ^207^Pb/^204^Pb) in cassiterites. These can be used for determining the U-Pb age of the mineral and the mineralisation processes [65; 112–116]. The approach can in general also be employed for tin objects since cassiterite is highly resistant to weathering, so that the mineral can be considered as a closed system with regard to the trace elements lead and uranium. In addition, the lead isotope ratios are not changed by the smelting process *per se*. Due to the low lead concentrations, however, there is a risk of anthropogenic lead contamination of tin metal during the smelting process by minerals accompanying the ore charge, by fuels used (e.g. charcoal) and by the smelting structures (furnaces, crucibles). Moreover, corrosion can contaminate tin posthumously by exchanging lead with the environment during burial. All these contamination sources can make it difficult, if not impossible, to determine the tin provenance by means of lead isotopes [65].

Nevertheless, lead isotope ratios were used in the past in sourcing tin in archaeological tin artefacts [16; 45; 59; 65; 76; 115]. By measuring the non-radiogenic ^204^Pb isotope and the uranogenic isotopes (^206^Pb, ^207^Pb) in tin metal, it is in principle possible to calculate the age of the parental cassiterite ores [114; 117]. If significant contributions of anthropogenic lead can be excluded, the lead isotope composition of the tin metal may be used to determine the provenance of the parental ore. Since cassiterite mineralisations throughout Eurasia were formed at different times in the Earth’s history this approach can help to narrow down potential tin ore sources. Of course, ores with the same formation age and the same common lead composition cannot be distinguished [115].

**Fig 11:**
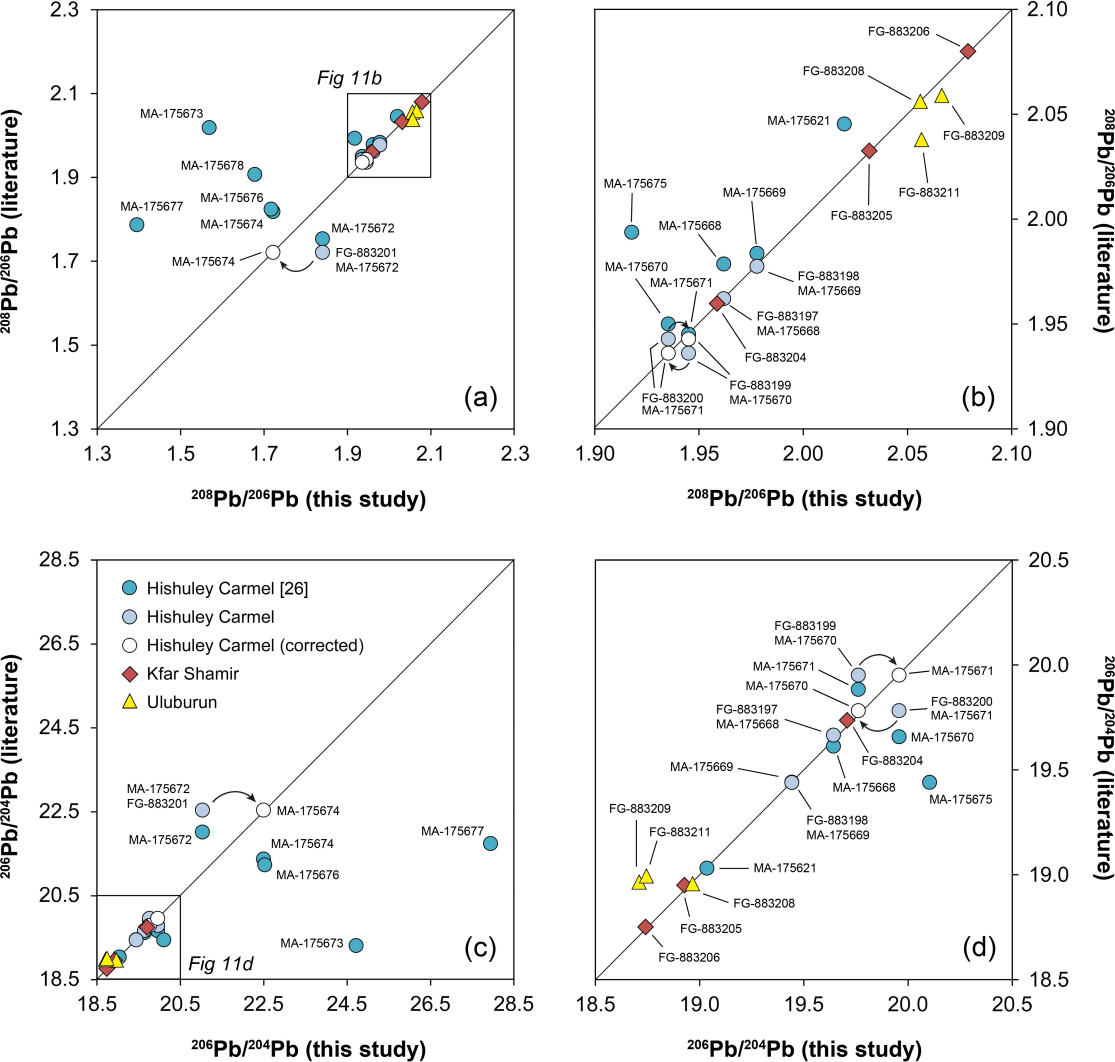
Comparison of lead isotope ratios determined in this study with data from the literature. (a–b) ^208^Pb/^206^Pb. (c–d) ^206^Pb/^204^Pb. If not stated in the legend (applies to all diagrams), the data was taken from Begemann et al. [62]. The white circles result from data-exchange of different samples (applies to data of Begemann et al. [62]) due to the recognised confusion in the sample set of Galili et al. [26]. Analytical uncertainties are smaller than the symbols (diagrams: D. Berger; data: B. Höppner; [26; 62]).

**Fig 12:**
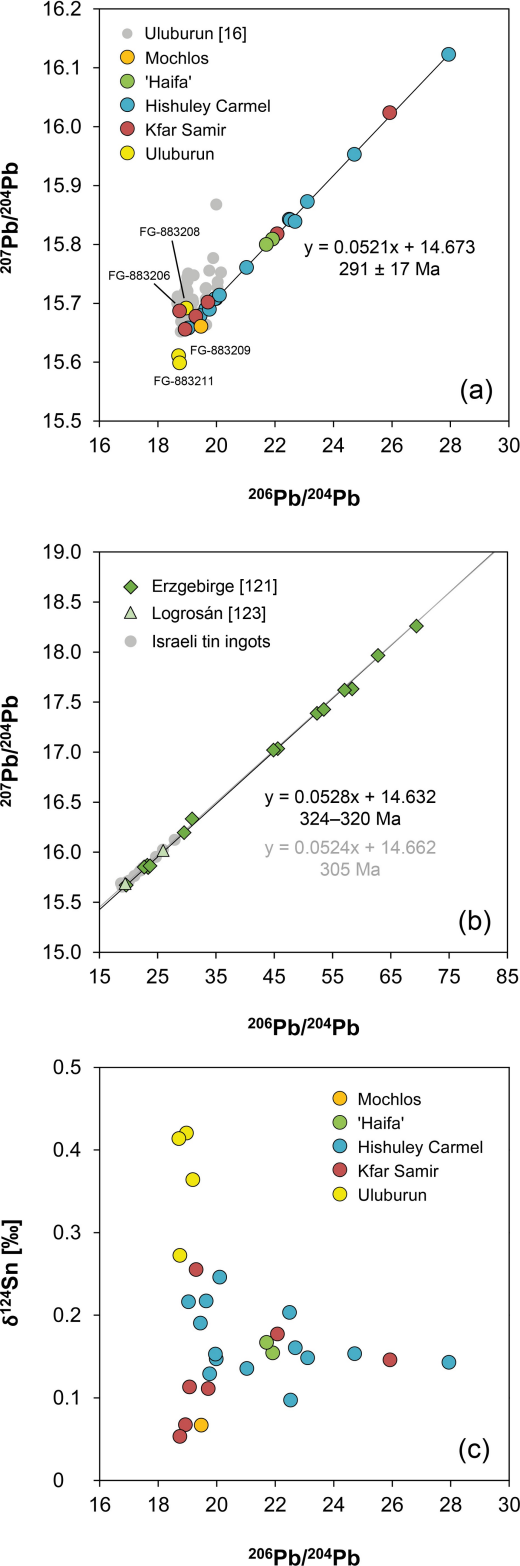
Lead isotope composition of the tin ingots examined in this study. (a) ^206^Pb/^204^Pb vs. ^207^Pb/^204^Pb compared with older data from Stos-Gale et al. [16] for the Uluburun objects. The data points of the Israeli ingots exhibit a linear trend which holds chronological information on the formation age of the original tin ores. The specified date of 291 ± 17 Ma was calculated as described in the text. (b) Isorchrons derived from lead isotope data of tin deposits in the Erzgebirge province (black) [121] and Logrosán (grey) [123] (no Pb-Pb data is available for the British tin sources). In (c) the ^206^Pb/^204^Pb ratio is plotted against the δ^124^Sn values. Analytical uncertainties are smaller than the symbols (diagrams: D. Berger; data: B. Höppner, G. Brügmann).

**Fig 13:**
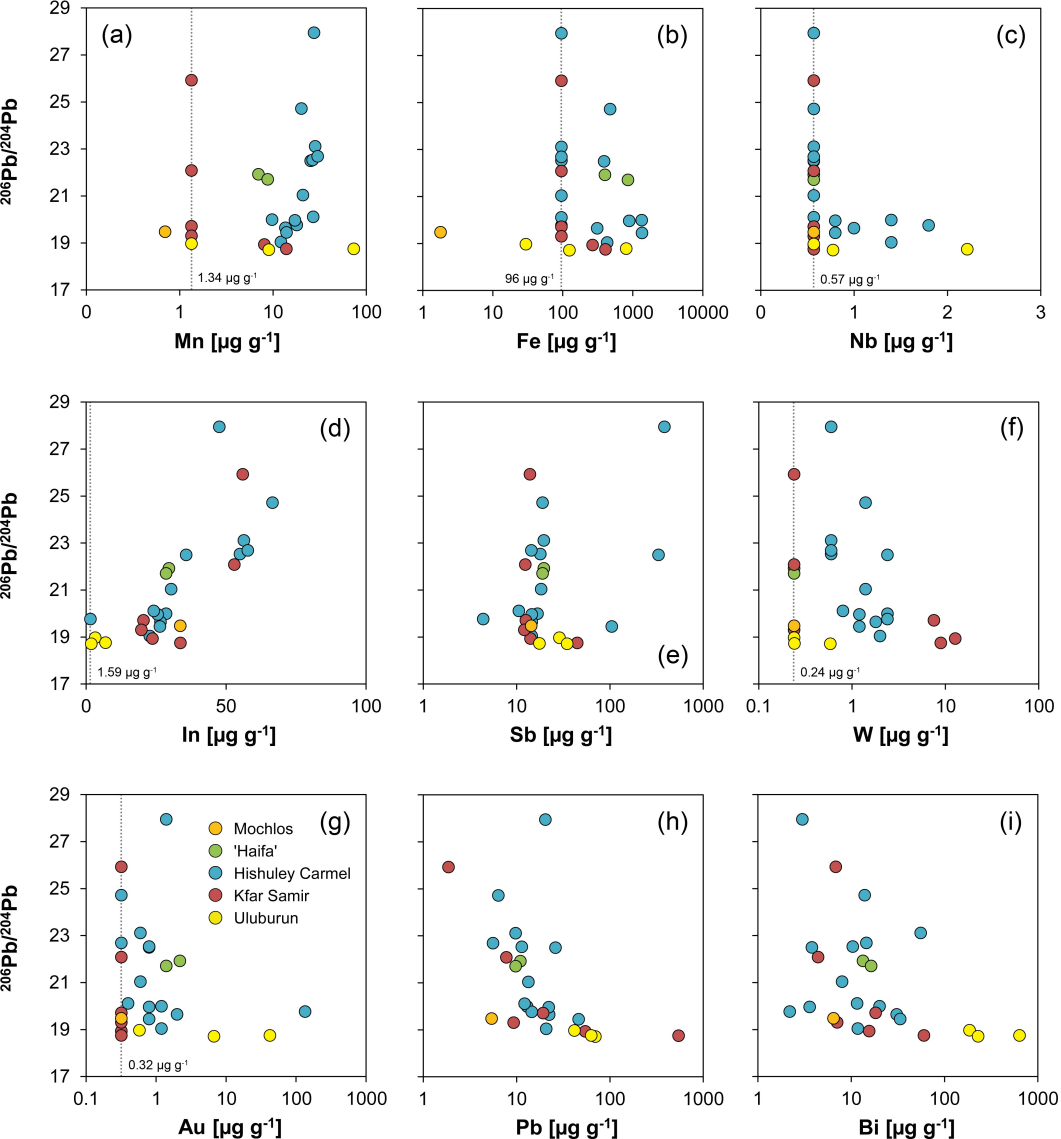
^206^Pb/^204^Pb isotope ratio of the tin ingots vs. the concentration of trace elements determined in this study. The dotted lines and specified values represent the limit of detection of the Q-ICP-MS, values for Mochlos are often lower due to the use of LA-Q-ICP-MS. Legend applies to all diagrams (diagrams: D. Berger; data: B. Höppner, N. Lockhoff).

The majority of the tin ingots have been analysed for their lead isotope ratios in the present study. The results are compiled in Figs 11–13 and Table 6. The data is also contrasted with the results of previous studies that analysed exactly the same samples or different aliquots of the same ingots as we did [16; 26; 62]. Fig 11 reveals a good agreement of our ^208^Pb/^206^Pb data with those of Begemann and co-workers [62], even in the case of the Hishuley Carmel ingots, of which we have analysed the duplicate samples previously studied by Stos-Gale et al. [16] and Galili et al. [26] (Fig 11A and 11B). A slight difference was observed for the ^206^Pb/^204^Pb isotope ratio, probably because of the lower analytical precision of the ^204^Pb isotope measurement that was achievable at that time (Fig 11C and 11D). Isotope ratios in samples MA-175670 (= HC1111/3; T3) and MA-175671 (= HC1111/4; T4) deviate significantly from those of Begemann et al.’s [62] samples FG-883199 (= 1111/3) and FG-883200 (= 1111/4) which were taken from the same ingots. As already suggested by the chemical data described above, this can most likely be attributed to sample mix-up of the duplicate samples MA-175670 and MA-175671 (T3 ↔ T4). If the values are interchanged, the data coincides perfectly (Fig 11B, white circles). There seems to be another mix-up with samples MA-175672 (= HC1111/5; T5) and FG-883201 (= 1111/5) because the values do not match even though the analysed samples should come from the same object. If the ratios from MA-175674 (= T8) are used, then we get again a perfect match (Fig 11A and 11C, white circles). From these results, we can conclude that the lead isotope composition in the individual ingots is homogenous, at least in the case of Hishuley Carmel, because measurements of duplicate samples yielded the same isotope values. The conclusion of Clayton [65], who found British tin ingots to be isotopically heterogeneous, is not supported.

**Table 6:** Tin isotope composition (δ^124^Sn, 2SD) and lead isotope ratios of all samples from this study.

Site	Lab. no.	δ^124^Sn	2SD	^208^Pb/ ^206^Pb	2SD	^207^Pb/ ^206^Pb	2SD	^206^Pb/ ^204^Pb	2SD	^208^Pb/ ^204^Pb	2SD	^207^Pb/ ^204^Pb	2SD
Mochlos	MA-145558a	0.09	0.01	n. a.		n. a.		n. a.		n. a.		n. a.	
	MA-145558b	0.07	0.02	1.9743	0.0001	0.80435	0.00002	19.470	0.001	38.439	0.003	15.661	0.001
Haifa	MA-175618	0.15	0.01	1.7599	0.0001	0.72136	0.00003	21.916	0.001	38.570	0.006	15.809	0.001
	MA-175619	0.17	0.02	1.7782	0.0001	0.72796	0.00001	21.704	0.002	38.595	0.015	15.800	0.001
Hishuley Carmel	MA-175620	0.15	0.01	1.9291	0.0001	0.78602	0.00001	19.985	0.002	38.552	0.009	15.708	0.003
	MA-175621	0.22	0.03	2.0197	0.0001	0.82261	0.00003	19.035	0.001	38.444	0.009	15.658	0.001
	MA-175668	0.22	0.01	1.9618	0.0001	0.79885	0.00004	19.642	0.002	38.534	0.013	15.691	0.002
	MA-175669	0.19	0.01	1.9778	0.0001	0.80648	0.00004	19.442	0.002	38.452	0.008	15.679	0.001
	MA-175670	0.15	0.01	1.9353	0.0001	0.78708	0.00001	19.957	0.002	38.622	0.009	15.708	0.002
	MA-175671	0.13	0.01	1.9450	0.0002	0.79401	0.00009	19.761	0.002	38.434	0.001	15.690	0.001
	MA-175672	0.14	0.003	1.8403	0.0001	0.74944	0.00002	21.030	0.003	38.701	0.008	15.761	0.001
	MA-175673	0.15	0.02	1.5680	0.0001	0.64551	0.00003	24.713	0.002	38.757	0.004	15.953	0.001
	MA-175674	0.20	0.01	1.7213	0.0002	0.70436	0.00004	22.492	0.005	38.716	0.031	15.843	0.004
	MA-175675	0.25	0.02	1.9177	0.0001	0.78169	0.00001	20.103	0.001	38.551	0.001	15.714	0.002
	MA-175676	0.10	0.03	1.7172	0.0001	0.70330	0.00005	22.525	0.001	38.680	0.001	15.842	0.001
	MA-175677	0.14	0.02	1.3953	0.0001	0.57712	0.00005	27.938	0.001	38.981	0.001	16.123	0.002
	MA-175678	0.15	0.01	1.6779	0.0001	0.68693	0.00001	23.108	0.005	38.772	0.001	15.873	0.002
	MA-175679	0.16	0.02	1.6980	0.0001	0.69826	0.00001	22.684	0.001	38.507	0.001	15.839	0.001
Kfar Samir south	FG-883202	0.11	0.002	n. a.		n. a.		n. a.		n. a.		n. a.	
	FG-883204	0.11	0.03	1.9587	0.0001	0.79672	0.00001	19.708	0.002	38.602	0.001	15.702	0.003
	FG-883205	0.07	0.01	2.0317	0.0001	0.82714	0.00001	18.928	0.001	38.456	0.004	15.656	0.001
	FG-883206	0.05	0.03	2.0791	0.0001	0.83700	0.00002	18.741	0.001	38.965	0.005	15.687	0.001
	MA-176924	0.15	0.02	1.4971	0.0001	0.61820	0.00002	25.922	0.001	38.808	0.001	16.024	0.001
	MA-176925	0.18	0.02	1.7512	0.0001	0.71650	0.00001	22.077	0.001	38.662	0.004	15.818	0.001
	MA-176926	0.26	0.01	1.9948	0.0002	0.81246	0.00003	19.297	0.001	38.493	0.001	15.678	0.002
Uluburun	FG-883208	0.42	0.01	2.0562	0.0001	0.82735	0.00001	18.967	0.001	38.998	0.006	15.692	0.001
	FG-883209	0.41	0.01	2.0665	0.0001	0.83439	0.00001	18.710	0.001	38.664	0.004	15.611	0.001
	FG-883210	0.36	0.01	n. a.		n. a.		n. a.		n. a.		n. a.	
	FG-883211	0.27	0.02	2.0568	0.0001	0.83215	0.00002	18.745	0.001	38.554	0.007	15.599	0.001

Samples tagged with ‘n. a.’ were not analysed for their lead isotope ratios (data: G. Brügmann, B. Höppner).

When comparing our dataset with those of Stos-Gale et al. [16] and Galili et al. [26] for the same specimens (the latter reproduced data for T1–T5 from the former and published new data for T6–T13), there is no match in the different isotope ratios. The best fit is observed for sample MA-175669 (= HC1111/2; T2) which indicates only a slight offset of the ^208^Pb/^206^Pb isotope ratio (Fig 11B and 11D). For all other samples, there is either good agreement for one isotope ratio (MA-175621, MA-175668, MA-175671) or no agreement at all. These differences are most likely not due to sample confusion since the values determined by us do not show up within the datasets of Stos-Gale et al. [16] and Galili et al. [26], respectively (Fig 11). We are currently unable to provide a comprehensive explanation, but contamination in the supplied samples with lead during chemical treatment or column-related fractionation effects occurring at this early stage of research into lead isotopes in tin are possible reasons (pers. comm. Z. Stos-Gale). A direct comparison of both datasets is therefore not possible, though this is ultimately not necessary for the interpretation of the available data.

The observed deviations of our data set with that of Begemann and colleagues [62] for the same samples from the Uluburun ingots could be due to contamination during corrosion. Apparently, the ingot still containing some metallic tin (FG-883208) is most consistent with the older data [62]. For the interpretations, we will use the new results of this study.

The isotopic composition of lead is used to narrow down the possible geological sources for the tin in the analysed ingots by dating the formation of their parental ores with the method described above. From the diagram with the ratios of the uranogenic lead isotopes ^206^Pb and ^207^Pb and the non-radiogenic lead isotope ^204^Pb (Fig 12A), it is apparent that basically all samples from Israel of our dataset plot along a linear trend (in contrast to the datasets of Galili et al. [26] showing unsystematic scattering). Pre- and post-depositional contaminations with anthropogenic lead are thus insignificant since such a trend cannot be produced by lead contamination of objects of different ages and found in different locations and environments. Exceptions are the ingots from the Uluburun shipwreck that probably suffered contamination as outlined above. The lead isotope composition of one of the Kfar Samir south ingots (FG-883206) could be compromised as well because it falls off the trend and has an unusually high lead concentration of 547 μg g^-1^ (Figs 8G and 12A and 12C). Its isotopic composition thus seems to be dominated by a foreign lead signature [115]. For this reason, we omitted the data of this sample and the Uluburun specimens from the discussion below. The same was done with the Mochlos ingot that seems to be out of the Israeli trend, either because it follows another trend or it suffered lead contamination, e.g. during corrosion.

The slope of the Israeli trend line can be used to calculate a geological model age [115; 117]. The lead of all samples that plot along this line–called isochron in geochemistry–was derived from cassiterites that formed at the same time in a specific geological environment from the same reservoir. If only the tin ingots from Hishuley Carmel are used to calculate the slope of the isochron then a value of 0.05220 is obtained. This relates to a geological age of 295 million years (Ma). If the samples of the other Israeli ingots are included (Fig 12A), the slope of the isochron is only slightly changed to 0.05213 ± 0.00038 (2SD) which gives a mean geological age of 291 Ma with a range between 308 and 274 Ma (2SD). This model age indicates that the cassiterite from the parent ore formed during the Variscan orogeny on the European continent. During this epoch the large tin deposits of Cornwall/Devon (295–270 Ma; [118–120]), the Erzgebirge province (320–280 Ma; [109; 116; 121]) and the Iberian peninsula (336–280 Ma; [90; 122–123]) were formed. A similar crystallisation age was reported for the tin mineralisation of the French Massif Central (317–298 Ma; [104; 124]) and Brittany (320–315 Ma; [104]) as well as for Sardinia (307–289 Ma; [125–126]). Thus, based on the lead isotopic composition, all these deposits could have provided the tin for the Israeli tin ingots analysed. To reinforce this conclusion, two isochrons from tin-related geological formations in the Erzgebirge province and the Logrosán deposit (Spain) are plotted in a diagram together with data of the tin (Fig 12B). They show similar slopes and are very close to the isochron of the tin ingots.

By contrast, central Asian, Indian and north African tin deposits can be excluded due to their much younger formation ages of ~80 Ma (Hindu Kush, Afghanistan) and ~100 Ma (Pamir, Tadzhikistan) or their much older ages between 650 and 530 Ma (eastern Desert, Egypt) and 1500 and 700 Ma (India) [115; 127–130]). Similarly, the mineralisations of Kestel and Hisarcık are too young as they formed during the Alpine orogeny between 20 and 2 Ma [131–132]. Finally, the tin occurrences in the Zagros Mountains (Deh Hosein), in the Mourne Mountains and the Slovak Ore Mountains are all excluded as well as they formed 230–180 Ma, 60–50 Ma or 150–120 Ma ago [133–135]. The lead isotope composition of the Israeli tin ingots thus strongly supports the idea of a tin origin from a European deposit.

This conclusion seems not to apply to the singular ingot from Mochlos. As stated above, its lead isotope ratios are not in perfect trend with the Israeli objects (Fig 12A). The deviation from the trend is admittedly not substantial, but since the isotopic composition (^206^Pb/^204^Pb = 19.47, ^207^Pb/^204^Pb = 15.661) is near the intersection point of isochrons with the same terrestrial common lead composition (^206^Pb/^204^Pb = 18.70, ^207^Pb/^204^Pb = 15.628; [136]), there is the likelihood that the Mochlos tin belongs to an isochron that characterises another geological age. Since the lead is less radiogenic than that in the Israeli ingots, it could lie on an isochron of tin ores that are younger than the European Variscan tin ores. Provided that no contamination in the lead occurred, this points to central Asian deposits such as those of Afghanistan and Tadzhikistan or to the tin source of Deh Hosein in the western Iranian Zagros Mountains. However, the latter is of polymetallic nature [133], and one would expect tin with high impurities (e.g. Cu, Fe, As, Sb) if such ores were used for smelting. Thus, the central Asian deposits could in fact have been the supplier for the Mochlos tin since other geologically young sources in the Mourne Mountains [101] and the Slovak Ore Mountains are not that likely, and the alleged tin mine of Kestel (geologically also very young) was not in operation any more during the 2^nd^ millennium BCE [137].

### 4.4. Tin isotope composition of the tin ingots

In order to discriminate among the European sources that were used for the tin ingots from Israel, the lead isotope data can be considered together with the artefact’s tin isotope composition. From the tin isotope systematics additional information regarding ore charges and metallurgical treatments can be inferred, particularly in combination with the trace elements.

**Fig 14:**
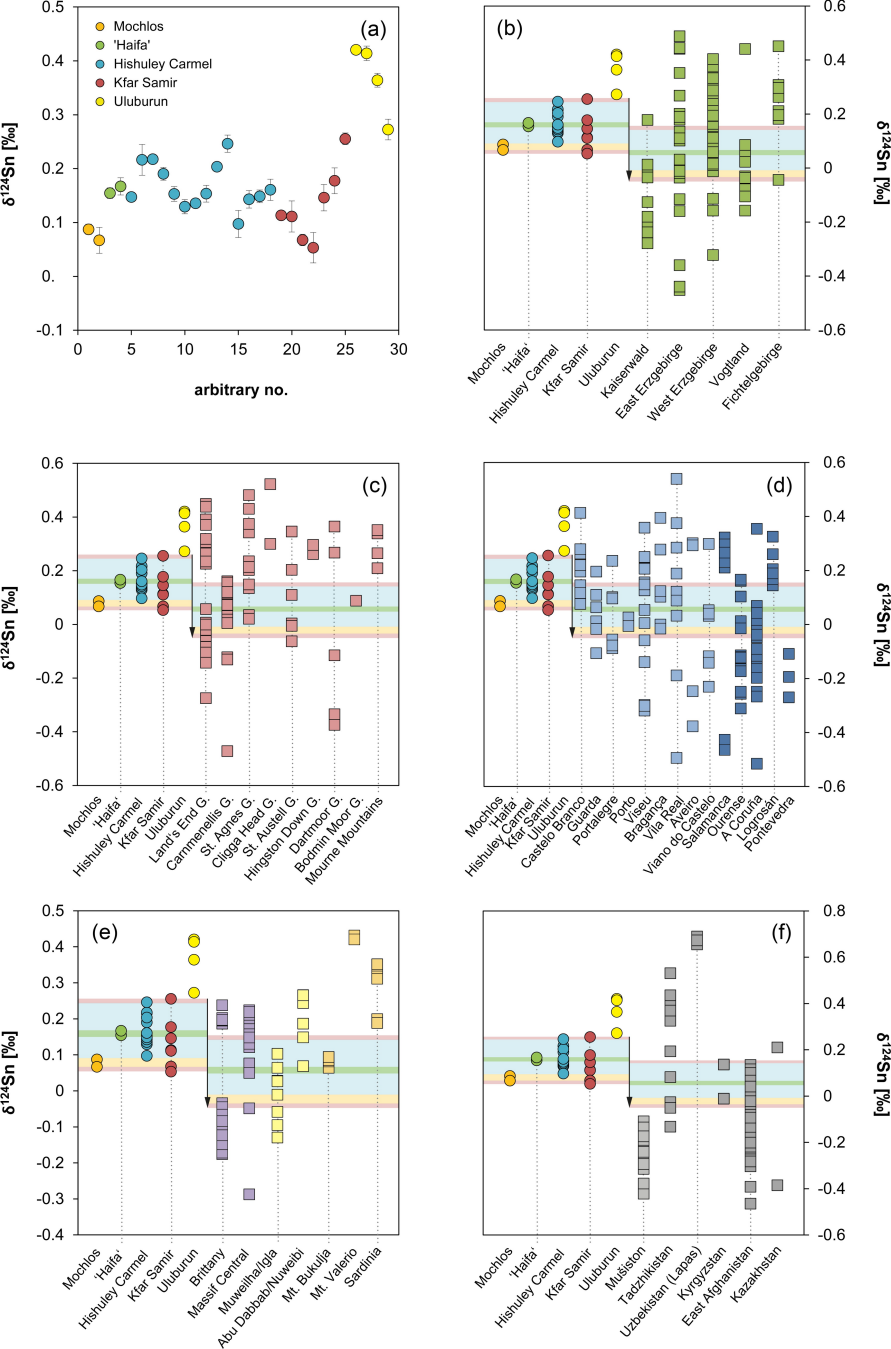
Tin isotope composition (δ^124^Sn) of the tin ingots examined in this study and comparison with tin ores. (a) Isotope composition of the ingots without taking the pyrometallurgical fractionation into account. (a–f) Comparison of ingots with ores from the Erzgebirge province (b), the British Isles (c), the Iberian peninsula (d), Brittany, the French Massif Central, Egypt, Sardinia, Mount Bukulja and Monte Valerio (e) and central Asia (f). The horizontal bars represent the variation in the tin and are lowered by the value 0.1 ‰ as pyrometallurgical impact on the right hand-side (indicated by the arrow) to yield the estimated original isotope composition of the ingots (cf. [69]). The colours of the bars correspond to the colours of the symbols used for the tin ingots. The numbering for samples in (a) corresponds to the sequence in Tables 6 and S2. Legend applies to all diagrams (diagrams: D. Berger; data: G. Brügmann, to be published numerically in the PhD thesis of J. Marahrens).

**Fig 15:**
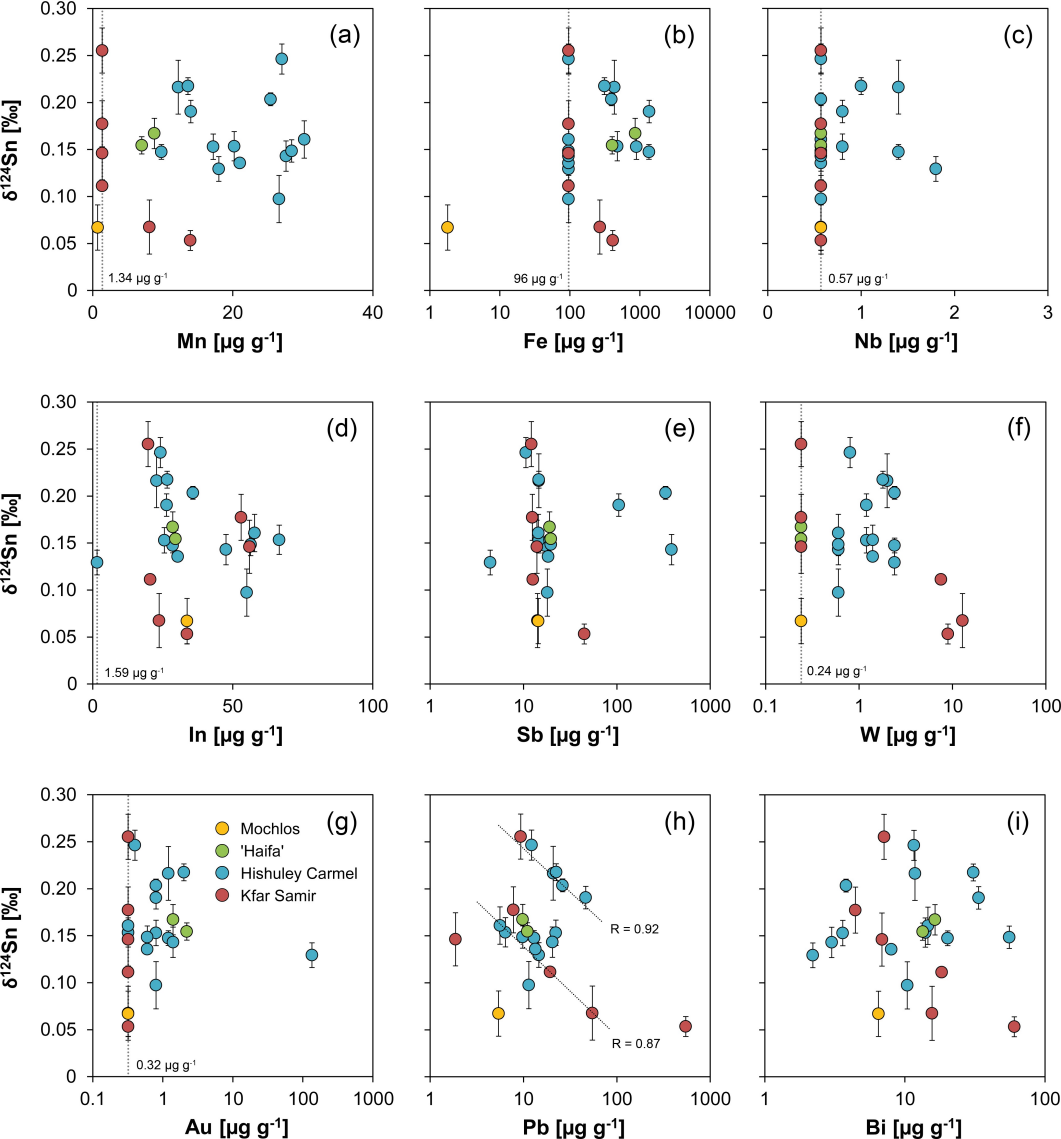
Tin isotope composition (δ^124^Sn) of the tin ingots vs. the concentration of trace elements determined in this study. The dotted lines and specified values represent the limit of detection of the Q-ICP-MS. Legend applies to all diagrams (diagrams: D. Berger; data: G. Brügmann, L. Lockhoff).

Fig 14A and Tables 6 and S2 summarise the tin isotope compositions of the tin ingots. Overall, only positive δ^124^Sn values (relative to our in-house standard) are observed that spread over a large range between 0.05 ± 0.03 ‰ and 0.42 ± 0.01 ‰. Individual sites, however, show a much smaller variation, and differences between them become discernible. The ingots can be divided into three groups (Fig 14A): one with high δ^124^Sn values of greater than 0.19 ‰, one with medium δ^124^Sn values ranging from about 0.12 to 0.18 ‰, and a third one with low δ^124^Sn values of less than 0.12 ‰. The large variation of the tin isotope ratios implies that the different groups of ingots–even within the same archaeological context–were produced from different tin ore charges (for comparison of actual data with older analyses see S1 File). Compared with trace elements, no distinct relationships are recognisable overall, but individual sites or groups of ingots with smaller variations in the tin isotopes reveal relationships with trace elements such as iron, antimony, tungsten, lead, bismuth, and indium (Fig 15).

The two ingots from Haifa have identical δ^124^Sn values within analytical uncertainties amounting to 0.15 ± 0.01 ‰ and 0.17 ± 0.02 ‰, respectively. Thus, these two ingots appear in fact to have belonged to a common find and archaeological context and were probably cast from the same metal batch as already suggested by their similar chemical and lead isotope compositions (Figs 12 and 15).

The isotopic composition of the fourteen ingots from Hishuley Carmel is on average identical with that of the Haifa samples (0.17 ± 0.08 ‰ vs. 0.16 ± 0.02 ‰). There is, however, a large variation of overall 0.15 ‰, and individual δ^124^Sn values range from 0.10 ± 0.03 ‰ to 0.25 ± 0.02 ‰ (Fig 14A and Tables 6 and S2).

The δ^124^Sn values fall into all three isotope groups defined above, but with the exception of one sample they have high and medium high isotopic ratios greater than 0.12 ‰ (Fig 14A). All data taken together, distinct relationships with trace elements cannot be recognised (Fig 15). However, the groups of ingots with the heaviest and the intermediate isotope compositions tend to follow two parallel trends with negative slopes for manganese, iron, indium, tungsten, bismuth and lead, which are particularly well defined with the latter with R = –0.90 and –0.80, respectively (Fig 15). These trends could imply that with increasing tin isotope ratios the metal concentrations decrease. This can occur when tin metal experiences a kind of refining process during which the metal is melted under oxidising conditions [96; 98]. Depending on the applied temperature, tin isotope ratios will increase during repeated melting and casting processes because of the possible loss of tin vapours or due to dross formation. At the same time, tin metal will oust elements as dross (mostly oxides), which are not completely soluble within its crystal lattice (e.g. elements forming intermetallics such as Fe and Mn) or which are less noble. Regardless of whether such operations were actually carried out and how they worked in detail, at least three different ore charges must have been used to produce these ingots. Their origin could be different tin sources, but they could as well stem from different mines or locations of the same deposit. In this regard, the two Haifa ingots were probably made from tin of the same mine as the Hishuley Carmel ingots with the intermediate isotopic composition as suggested by similar tin isotope ratios and trace element patterns (Fig 15).

**Fig 16:**
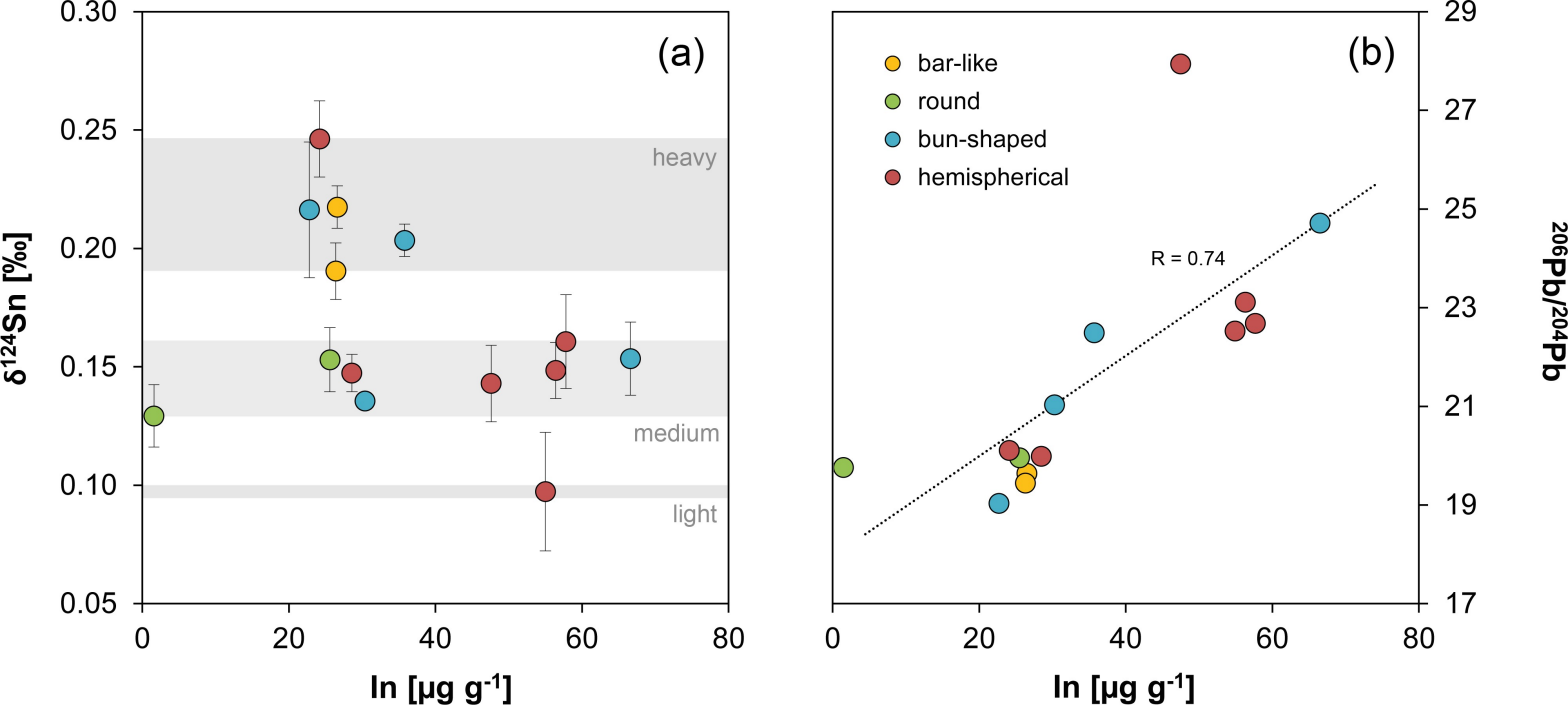
Tin and lead isotope composition of the different shaped ingots from Hishuley Carmel and their relationship with the indium contents. Note the strong correlation of the lead isotopes and indium (R = 0.74). Legend applies to both diagrams (diagram: D. Berger; data: G. Brügmann, B. Höppner, N. Lockhoff).

As shown by Galili and colleagues [26] the ingots of Hishuley Carmel have very different physical shapes varying from bar-like and round, hemispherical to bun-shaped (cf. Fig 4). The two bar-like and the two round shaped ingots each have very similar tin and lead isotope compositions (Fig 16). The two types of ingots have significantly different tin isotopic compositions where the bar-shaped ingots were produced from ores with heavy isotope composition, whereas round ingots derived from ores with the medium isotope composition. Although trace element concentration such as antimony, lead and indium differ among the two ingot types, this difference could be due to the variable depletion of these elements in the tin ingot depending on the temperature and redox conditions during the casting process as outlined above. However, hemispherically- or bun-shaped ingots have variable tin and lead isotopic compositions. They do not indicate a systemic relationship to any of the three groups defined by the tin isotope ratios (Fig 16) which implies that each ingot type was produced from a different charge of different tin ores. Hence, the physical shape of the ingots appears to contain no provenance information provided the recognised sample confusion does not carry weight here (all confused samples are accounted for).

Variation in the tin isotopes is even larger for the ingots from the Kfar Samir south site (Fig 14A and Tables 6 and S2). The data cover all three tin isotope groups with δ^124^Sn values ranging from 0.05 ± 0.03 ‰ to 0.25 ± 0.01 ‰ with an average composition of 0.13 ± 0.14 ‰. Compared to the averages of the ingots from Haifa and Hishuley Carmel, the tin from the Kfar Samir south assemblage has a slightly lighter tin isotope composition (Fig 14A, nos. 19–25) because of the predominance of the group with the lowest isotope composition (0.05 ± 0.03 ‰ to 0.11 ± 0.002 ‰). This group appears to have a differing chemical composition. It is richer in manganese, iron, lead, bismuth and tungsten and poorer in indium (Fig 15) than the remaining ingots from this ship. It is therefore likely that the tin ore for the production of the Kfar Samir south cargo came from different locations and at least from two different tin mines. Compared with the other sites, ores of the same deposits could have been used for the ingots with the medium high and high tin isotope composition, but a different one was certainly exploited for producing the Kfar Samir ingots with low tin isotope compositions.

The tin isotope composition from the corroded Mochlos ingot fragment was analysed in two samples. One sample represents the whitish surface crust (MA-145558a), the second one the brownish ingot core (MA-145558b). The isotope composition of both samples agree well within analytical errors (δ^124^Sn = 0.09 ± 0.01 ‰ and 0.07 ± 0.02 ‰); nonetheless the slight difference could perhaps indicate isotope fractionation during corrosion. As described above, the surface layer mainly consists of stannic oxide with tetravalent tin ions (Sn^4+^), whereas the core additionally contains mineralogical phases with divalent tin ions (Sn^2+^) such as romarchite and hydroromarchite (Figs 6 and 7A). Because tin is known to be a redox-sensitive element and fractionation factors have been shown to be different for tin phases of various oxidation states [138], the formation of different corrosion phases likely influenced the isotope composition of the samples. Further, more oxidised species (i.e. Sn^4+^-bearing minerals) of other redox-sensitive elements (Fe, Cu, Zn) have been shown to be isotopically heavier than less oxidised species (i.e. Sn^2+^-bearing minerals) or the metal phase [139]. This could explain the slightly different isotope ratios between the two Mochlos samples. The overall effect of corrosion on artefacts found in terrestrial environments, however, appears to be small as shown by comparative investigations on corrosion crusts and residual metal cores of tin and bronze objects [77; 81]. The mean isotope composition of the Mochlos ingot (δ^124^Sn = 0.08 ± 0.03 ‰) is thus assumed to reflect the composition of the original uncorroded tin. Provided this assumption is valid, the Mochlos tin belongs to the group with the lowest isotope ratios similar to those of the Kfar Samir south objects. According to the similar antimony, indium and gold concentrations, a production with tin ore from the same deposit would be suggested (Fig 15), but this contradicts the interpretation from the lead isotope systematics (see above).

The conclusions from the tin isotopes for the Greek and the Israeli ingots cannot be transferred to the Uluburun ingots. The artefacts from the wrecked Turkish ship are (almost) completely corroded as well, yet what distinguishes them from the Cretan artefact is their burial context: all items corroded in seawater instead of terrestrial soil. This led to the formation of a different corrosion assemblage consisting predominantly of abhurite (Sn_21_O_6_(OH)_14_Cl_16_) along with romarchite and stannic oxide (Figs 7B and 10). Although a detailed explanation is lacking at present, most of the corroded artefacts from seawater contexts analysed so far show enrichment in heavy tin isotopes between Δ^124^Sn = 0.05 ‰ and 0.30 ‰ relative to the uncorroded metal [81]. Isotope discrimination in seawater thus seems to be more pronounced than in soil. Although we cannot verify such a fractionation for the Uluburun ingots, because we could not find and analyse a reasonable amount of preserved tin metal, their extremely heavy isotope compositions ranging from of 0.27 ± 0.02 ‰ to 0.42 ± 0.01 ‰ (Fig 14A, nos. 26–29) suggest that the isotope ratios were altered during the corrosion process and do not reflect the original composition of the tin. This conclusion is supported by the significantly different isotopic compositions of two samples from ingot KW 203 taken from its corroded core (FG-883210) and the surface (FG-883211) (Fig 14A, nos. 28–29). This difference might be explained by the formation of tin corrosion products with different oxidation states, as already suggested for the Mochlos tin (Fig 7B). Because of all these findings, it is not possible to source the tin of the actual Uluburun ingots with the aid of tin isotopes. Fresh material is needed in order to overcome this problem.

However, the source of the tin in the remaining ingots can be discussed further as long as the change in the isotope ratios during the smelting process is taken into account. Smelting experiments with cassiterite recently carried out under prehistoric conditions demonstrated the loss of volatile tin species to induce a fractionation of tin isotopes. Relative to the ore, this caused heavier δ^124^Sn values in the tin metal by about 0.1 ‰ if 30% of the tin was recovered as tin metal [69]. We therefore have to consider this isotopic shift as ‘metallurgical impact’ when comparing metal artefacts with tin ores. Nevertheless, the experimental findings provide us with an important tool, namely that ores with heavier isotope compositions (in our case with higher δ^124^Sn ratios) than bronze or tin artefacts can be excluded as parental tin sources. The comparison of objects with each other is not significantly affected since isotope fractionation by smelting can be assumed to be more or less the same for all.

Fig 14B and 14F compare the ‘original’ isotope composition of the ingots (indicated as coloured bars)–i.e. the measured isotope composition minus the metallurgical impact of ca. 0.1 ‰ –with that of tin ores throughout Eurasia hitherto available from our database. Cassiterite from placers and lodes from the Erzgebirge region (Erzgebirge, Vogtland, Fichtelgebirge, Kaiserwald (Slavkovský les)), United Kingdom (Cornwall, Devon, Mourne Mountains), Brittany, the Massif Central, the Iberian peninsula (Portugal, Spain), Tuscany (Monte Valerio), Sardinia, Serbia (Mount Bukulja), Egypt, east Afghanistan (Hindu Kush), Uzbekistan (Lapas), Tadzhikistan (Mušiston, Pamir mountains), Kyrgyzstan and Kazhakstan are considered there. The figures clearly illustrate the principle difficulty in tin provenancing. Overall, the isotopic composition of cassiterite from the largest European tin provinces, namely Cornwall/Devon, the Saxon-Bohemian Erzgebirge and the Iberian peninsula, exhibits a wide variation and a significant overlap. The picture is somewhat differentiated when looking at single regions within these large tin provinces or even at single mines. Smaller variations and differing intervals can be recognised, which also applies to minor tin deposits such as those on Sardinia, at Monte Valerio, at Mount Bukulja or in the Mourne Mountains (North Ireland). The situation seems to be more promising with central Asian ores, but here our database is still lacking data from some major regions (e.g. Uzbekistan, Kazakhstan, Kyrgyzstan). For example, Afghan cassiterites from the Hindu Kush tend to be isotopically lighter compared for instance with Tadzhik tin ores from the Pamir and the data overlap very little (Fig 15F).

Given that, a positive assignment of a tin or bronze artefact to a specific tin mineralisation is not possible because of data overlap. The geographical location of the initial tin source can only be approximated by excluding those ore bodies whose isotope compositions differ from that of the artefacts. In the case of the Mochlos ingot this means that deposits having δ^124^Sn ratios of greater than 0 ‰, such as those from the French Massif Central and the smaller deposits of Monte Valerio, Sardinia, Mount Bukulja and the Mourne Mountains can be excluded if we consider a fractionation of Δ^124^Sn = 0.1% (for 30% tin recovery) during the smelting process (Fig 15E). Most of these deposits were made less likely because of their formation age (see above) or their ineptitude for Bronze Age tin exploitation [43; 45]. For the same reason, the other European mineralisations from the Erzgebirge, Cornwall/Devon, the Iberian peninsula and Brittany as well as those from Egypt would be excluded. Of the remaining possible sources, we do not have tin isotope data from Deh Hosein, but, as explained above, the polymetallic character of this deposit speaks against its use for the Mochlos tin. We therefore consider the central Asian tin deposits in the eastern part of Afghanistan and in Tadzhikistan as the most reasonable sources for the tin from Mochlos, not least because their tin isotope systematics (that we determined so far) agree well with the that of the ingot. In Tadzhikistan, cassiterite from Takfon (near the Mušiston deposit) and from Ghilnoye in the Pamir mountains shows the best match within our dataset (Fig 15F). One sample from Kyrgyzstan also shows a match, but we have just one data point that is not statistically significant. There are also more tin sources along the borderline of the Herat and Farah provinces in West Afghanistan and in the central Afghan Daykundi province [54; 56–57] from which we had no material to be analysed.

The situation is exactly the other way round for the Israeli ingots. The lead isotope ratios strongly suggest a European tin source and exclude Asian and African deposits. Because of data overlap with the ores and larger variations within the finds, a couple of mines have to be taken into consideration for the Hishuley Carmel and Kfar Samir south ingots. The list of potential suppliers is also long for the two analysed ingots from Haifa. On the basis of our tin isotope data of ores the most probable candidates are the eastern and western parts of the Erzgebirge, cassiterites from the Carnmenellis and St. Austell granites in Cornwall and several regions on the Iberian peninsula (Fig 15 and Table 7). Brittany and the minor tin occurrences at Monte Valerio, Sardinia and the Mourne Mountains are very unlikely sources for the tin artefacts as their isotope characteristics are distinctively different from those of the Haifa ingots (Fig 15).

**Table 7:** Possible tin sources for the tin ingots as inferred from the tin isotope data and after accounting for the lead isotope ratios.

Country	Mochlos	Hishuley Carmel	Haifa	Kfar Samir south
DE/CZ		*Vogtland*	*Vogtland*	*Vogtland*
		East Erzgebirge	East Erzgebirge	East Erzgebirge
		West Erzgebirge	West Erzgebirge	West Erzgebirge
GB		*Land’s End granite*		*Land’s End granite*
		Carnmenellis granite	Carnmenellis granite	Carnmenellis granite
		St. Austell granite	St. Austell granite	St. Austell granite
PT^†^		Guarda province	Guarda province	Guarda province
		Viseu province	Viseu province	Viseu province
		Bragança province	Bragança province	*Bragança province*
			Vila Real province	
			Viano do Castello	
ES^†^		Ourense province		Ourense province
			A Coruña province	
FR		*Massif Central*	*Massif Central*	*Massif Central*
TJ	Takfon			
	Ghilnoye			
AFG	Hindu Kush			
KG	*Kyrgyzstan*			

Those deposits that are most likely the sources are underlined, those that are less likely but not excludable are italicised. Because of the missing archaeological evidence (see text) the Iberian ores designated by an † are not likely the sources for the Mediterranean tin ingots.

All of the sources for the Haifa tin could have been used for the production of the ingots from Hishuley Carmel and Kfar Samir. If one assumes a common origin for the ingots in one cargo because they were found very close together at one site, this would exclude tin deposits that do not completely cover the range of isotope ratios observed (Land’s End granite in Cornwall, the Castelo Branco region in Portugal or Cerro de San Cristóbal, Logrosán in Spain [140–141]; Fig 15 and Table 7). However, it is possible that tin was collected from various mining areas in large centres or trading ports such as Ugarit, Byblos, Tyre, Cyprus, Cornwall etc. [18], and thus individual ships transported cargoes containing tin ingots of mixed provenance. The different groups and shapes of ingots observed at Hishuley Carmel and Kfar Samir might reflect this situation. In this case, the different isotopic groups have to be discussed separately and the interpretation is further complicated when the tin isotope composition is considered alone. Yet, by including the trace element patterns of the Mediterranean tin ingots, the potential sources can be confined further. Because the elemental composition is quite similar to those of the Salcombe ingots (Fig 8), and the latter were certainly made from Cornish or Devonian tin ores [94], a British provenance of the tin from Israel is currently the most reasonable. The comparably high indium concentration in the ingots that is a typical feature of Cornish cassiterites might be the most helpful indication. On the other hand, the very low indium contents of the Uluburun ingots as well as their differing elemental pattern in general indicate a source of ore other than southwest England. It would be tempting to infer geologically very old tin deposits (such as Egypt or India) from the existing lead isotope data (literature data and own data; Fig 12A, grey and yellow dots), but because of the corroded nature of the objects, neither the chemical data nor the lead and tin isotopic compositions provide any reliable information on their provenance. For the Israeli ingots the situation is much better, and all parameters taken together indicate that the ingots (except for the isotopically lowest group of Kfar Samir south) were produced from tin ores of the same deposit, albeit different ore charges or ores from different mines were certainly used. Given this and the proximity of the sites, it even seems possible that the suspected ships belonged to a fleet that sank for example during a single storm event.

## 5. Conclusions

The archaeometallurgical examination of 27 LBA tin ingots (1530–c. 1300 BCE) from five sites in the eastern Mediterranean area enables the localisation of the potential suppliers of the tin ores by means of chemical and isotopic analyses for the first time. The lead isotope composition of the tin is the most important fingerprint in this regard. It clearly identifies European deposits as tin sources for the Israeli ingots because the Pb-Pb model age of the tin of about 290 Ma links the Variscan orogenic belt to the parental tin ores used for the production of the tin ingots. The tin isotope composition helps to further narrow down the tin origin, and in combination with trace elements it points to Cornish tin ores (possibly from Carnmenellis granite area) as the most likely sources. However, other European sources, such as the Erzgebirge province or the French Massif Central, cannot be excluded categorically. Thus, additional systematic analytical research on the isotopic and chemical composition of tin ores and tin objects in combination with experiments on the behaviour of trace elements during smelting is needed in order to verify these conclusions. If the analytical data were considered alone, Iberian tin ores would also represent potential sources for the Mediterranean tin ingots. Preliminary arguments against these sources provide the very low concentrations of indium and antimony in both experimental smelted tin metal from Iberian ores [69] and a late Bronze Age tin foil from Huelva, Spain (unpublished). But also because of the sparse evidence of contacts between the people of the eastern Mediterranean and the western and north-western regions of the Iberian peninsula in the second half of the 2^nd^ millennium BC, the exploitation of these ores to produce the ingots is unlikely from an archaeological point of view [47; 142–144]. In particular, there is a lack of evidence for the existence of elites or communities who were able to organise and control far-reaching and long-standing trade networks. The same is true for Cornwall and Devon although there is at least some weak material evidence for contacts between the British Isles and the central Mediterranean area (especially Sicily) via the lands in between [133]. Direct contacts between the British Isles and the Eastern Mediterranean are not assured at present, while inter-regional and international trade networks between the latter and northern and central Europe seem to be well documented for the second half of the 2^nd^ millennium BCE [145–148]. These connections are mainly documented by the trade with Baltic amber, Mesopotamian or Egyptian glass beads, and iconographic evidence, but it was Muhly [47; 142] who brought forward and developed the idea of the trade with tin. Although we cannot provide any new archaeological proof of that, the results of our analytical study might shed new light on this old question on tin sources. The Israeli tin ingots could be examples for emerging tin networks between north-western Europe and the eastern Mediterranean area–probably via the Greek mainland under the Mycenaean regency–that could have persisted some hundred years.

The conclusions drawn above appear to be in conflict at first glance with the textual evidence from Kültepe/Kaneš and Mari dating from the early 2^nd^ millennium BCE (around 20^th^–18^th^ century BCE). These texts point to tin sources somewhere to east of Anatolia and the Levant, and especially those from Mari suggest that a trade of tin to Crete is conceivable [47]. Moreover, connections between Crete and the Near East are well-documented [149–150], and Muhly [47] and Pigott [55] saw no alternative to an eastern origin of the Minoan tin. So, this should also hold true for the mid-16^th^ century tin ingot from Mochlos, as it seems to be suggested by its lead isotope composition. Further evidence in this regard comes from the Mochlos settlement itself, which yielded several beads of lapis lazuli that were hidden inside an ivory *pyxis*. The beads are contemporary with the tin and were found in House A.2 (‘the House of the Lady with the Ivory Pyxis’) next door to Building B.2 where the ingot was found [151–152]. The material clearly connects Mochlos with central Asia, especially Afghanistan [153], and it is reasonable that the tin from that area reached Crete in the same way as earlier in the first half of the 2^nd^ millennium BCE.

But do the Assyrian and Sumerian texts also apply to the later centuries of the 2^nd^ millennium BCE? One has to bear in mind that these texts were written some five hundred years before the Israeli (and the Turkish) ingots were produced. It is therefore not too daring to propose a change in eastern Mediterranean trade networks from the later second half of the 2^nd^ millennium BCE onwards on the basis of our results. This has already been suggested by Kassianidou [35] in connection with the Israeli tin ingots having Cypro-Minoan inscriptions. She argued for a predominant position of Cyprus in the copper and tin trade, probably favoured by the collapse of trade routes to the east due to the decline of the Levantine states. A consequence of the interruption of the tin trade was that new tin resources had to be sought, which could be found on the European continent and even on the British Isles. It is no accident that the shift in the tin trade from the Near East to Europe and Cornwall in particular, documented by the isotopic and chemical evidence, corresponds to the demise of the Minoans and the rise of the Mycenaeans ca. 1430 BCE. Unlike the Minoans, the Mycenaeans sailed west and established several trading ports in southern Italy, Sicily, Sardinia and south Iberia, which served as gateways to new trading routes to Britain and the European interior [154]. It is uncertain, however, how the tin cargos of the Uluburun and Gelidonya wrecks fit in this picture. They are heavily weathered, and at present it can only be asserted that the chemical composition of the Uluburun tin differs from the Israeli and the Mochlos ingots and compares to Sardinian tin artefacts (and ores). It is possible that the oxhide shape of many of the ingots as well as the incised marks [18] contain additional information, but whether or not these details indicate a different origin of the tin remains an open question. Fortunately, samples of uncorroded metal should provide good prospects for future studies.

Although many questions remain unanswered and new ones arose, the integrated approach of using trace elements, tin and lead isotopes turns out to be a promising tool for provenancing and fingerprinting ancient tin objects. It should be followed up by future archaeometallurgical research in order to unravel the enigma of BA tin. In this form, our study should stimulate new discussions on the provenance of tin of the Eurasian BA rather than postulating an origin from a specific deposit.

## Supporting information

S1 Table Conditions and parameters of the analytical facilities employed in the study. (XLSX)

S2 Table Full tin isotope data (δ values) and standard deviations of all samples analysed in this study. (XLSX)

S3 Table List of locations for the cassiterite samples used for comparison with the tin of the ingots in the study. (XLSX)

S1 File Comparison of tin isotope data of this study with data of previous studies and evaluation. (DOCX)
