# Supplementary material for: Isotope systematics and chemical composition of tin ingots from Mochlos (Crete) and other Late Bronze Age sites in the eastern Mediterranean Sea: An ultimate key to tin provenance?
Source: PLoS One. 2019 Jun 26;14(6):e0218326. doi: 10.1371/journal.pone.0218326 (PMC6594607; doi:10.1371/journal.pone.0218326)
Supplement: S1 File — (DOCX) [file pone.0218326.s004.docx]

**S1 File.** Comparison of tin isotope data of this study with data of previous studies and evaluation.

An earlier study carried out by Gillis and co-workers [32] (data also reported in [33]) also observed large differences among four ingots from Hishuley Carmel that we re-analysed in this study (MA-175674/75, MA-175678/79). Their data (δ^122^Sn/^116^Sn) suggest an overall isotopic discrimination of –0.08 ‰ u^-1^ whereas our study indicates a fractionation of +0.05 ‰ u^-1^ (S4 Table; [32]). Thus, even though the study used the same Puratronic tin metal as an in-house standard, the isotopic compositions are systematically different. We do not have an explanation for that diametrical result, but heterogeneities within the large samples of the ingots (ca. 50 g) are highly unlikely. Large variations within individual archaeological (bronze) artefacts have not been observed so far (own analyses, to be published). Even if one assumes that single ingots were cast from more than one metal batch (as observed for copper ingots; e.g. [99]), such a large overall isotope fractionation would not be expected. The data published earlier by Gale [31] for two of the Hishuley Carmel ingots are in good agreement with the values reported here (S2 Table; S4 Table). Furthermore, Nowell and colleagues [72] produced a dataset for another Hishuley Carmel ingot that was not available to us (Galili 3 = 3 = T9 in [26]). Their measured isotope ratio (^122^Sn/^116^Sn) falls well into our data range (S2 Table; S4 Table).

Gillis and co-workers [29] also analysed one of the Kfar Samir ingots (G21 = MA-176925). They reported a much lighter tin isotope composition (δ^122^Sn/^116^Sn = –2.20 ± 0.05 ‰) than that of the ingots from Hishuley Carmel (S4 Table), but again, this is not in agreement with the data we measured (δ^122^Sn/^116^Sn = 0.29 ± 0.02 ‰; S4 Table). The isotope composition given by Gale [31] for another Kfar Samir south ingot (81/608-5 = FG-883204) is also too light, whereas Nowell and colleagues [72] reported a distinctly heavy isotope composition for an ingot if compared to our data of the whole assemblage (S2 Table; S4 Table, 81/608-5, Galili 20). Since we did not have access to Nowell et al.’s sample it is difficult to evaluate the significance of their result.

References

31. Gale NH. The isotopic composition of tin in some ancient metals and the recycling problem in metal provenancing. Archaeometry. 1997; 39(1): 71–82.

32. Gillis C, Clayton RE, Pernicka E, Gale NH. Tin in the Aegean Bronze Age. In: Polinger Foster K, Laffineur R, editors. Metron: measuring the Aegean Bronze Age, Proceedings of the 9^th^ international Aegean conference New Haven, Yale University, 18–21 April 2002. Aegaeum 24. Liège: Université de Liége; 2003. pp. 103–110.

33. Gillis C, Clayton R. Tin and the Aegean in the Bronze Age. In: Tzachili I, editor. Aegean metallurgy in the Bronze Age: proceedings of an international symposium held at the University of Crete, Rethymnon, Greece, on November 19–21, 2004. Athens: Ta Pragmata; 2008. pp. 133–142. Athens.

72. Nowell G, Clayton RE, Gale NH, Stos-Gale ZA. Sources of tin: is isotopic evidence likely to help? In: Bartelheim M, Pernicka E, Krause R, editors. Die Anfänge der Metallurgie in der alten Welt. Forschungen zur Archäometrie und Altertumswissenschaft 1. Rahden/Westf.: Marie Leidorf; 2002. pp. 291–302.

99. Hauptmann A, Maddin R, Prange M. On the structure and composition of copper and tin ingots excavated from the shipwreck of Uluburun. Bulletin of the American Schools of Oriental Research. 2002; 328: 1–30.

**S4 Table.** Tin isotope composition (δ^122^Sn/^116^Sn, 2SD) of several tin ingots from the present study contrasted with data of preceding studies. Values from the literature have been re-calculated according to the specification in the respective publications that used the same Puratronic in-house standard. Italicised values are only estimates due to missing numerical values in the literature (data: G. Brügmann; [31–32, 72]).

| **Site** | **Lab. no. (CEZA)** | **Original designation** | **δ^122^Sn/^116^Sn** | **2SD** | **δ^122^Sn/^116^Sn** | **2SD** | **Reference** |
| --- | --- | --- | --- | --- | --- | --- | --- |
|  |  |  | (This study) | | (Literature) | |  |
| Haifa | No sample | CMS6 (Dor) | n. a. |  | *-0.74* |  | [31] |
| Hishuley Carmel | MA-175668 | HC1111/1 | 0.36 | 0.02 | *0.33* |  | [31] |
|  | MA-175669 | HC1111/2 | 0.31 | 0.03 | *0.42* |  | [31] |
|  | MA-175674 | G8 = T8 | 0.33 | 0.02 | -0.49 | 0.04 | [32] |
|  | MA-175675 | G13 = T10 | 0.40 | 0.02 | -0.38 | 0.04 | [32] |
|  | MA-175678 | G15 = T13 | 0.24 | 0.03 | -0.31 | 0.05 | [32] |
|  | MA-175679 | G11 = T9 or T14? | 0.24 | 0.03 | 0.02 | 0.03 | [32] |
|  | No sample | Galili 3 = T9 | n. a. |  | 0.19 |  | [72] |
| Kfar Samir south | FG-883204 | 81/608-5 | 0.18 | 0.04 | *-0.15* |  | [31] |
|  | MA-176925 | G21 | 0.29 | 0.02 | -2.20 | 0.05 | [32] |
|  | No sample | Galili 20 | n. a. |  | 0.55 |  | [72] |
| Uluburun | No sample | KW2235 | n. a. |  | 0.47 | 0.05 | [32] |
|  | No sample | KW519 | n. a. |  | 0.01 | 0.02 | [32] |
|  | No sample | KW392 | n. a. |  | -0.81 | 0.01 | [32] |
